# A comprehensive comparison of the safety and efficacy of drugs in the treatment of idiopathic pulmonary fibrosis: a network meta-analysis based on randomized controlled trials

**DOI:** 10.1186/s12890-024-02861-w

**Published:** 2024-01-27

**Authors:** Xiaozheng Wu, Wen Li, Zhenliang Luo, Yunzhi Chen

**Affiliations:** Department of Preclinical medicine, Guizhou University of Traditional Chinese Medicine, Guiyang, 510025 China

**Keywords:** Drug, Idiopathic pulmonary fibrosis, Safety, Efficacy, Meta-analysis

## Abstract

**Objective:**

Randomized controlled trials(RCTs) of multiple drugs for Idiopathic pulmonary fibrosis(IPF) have been reported and achieved a certain degree of efficacy, however, the difference in safety and efficacy of them for IPF is not yet well understood. The aim of this network meta-analysis is to assess their safety and efficacy in the treatment of IPF and differences in this safety and efficacy comprehensively.

**Methods:**

The PubMed, EMbase, CENTRAL and MEDLINE were retrieved to find out the RCTs of drugs in the treatment of IPF. The retrieval date is from construction to November 10, 2022. Stata 14.0 and RevMan 5.3 was used for statistical analysis. Registration number: CRD42023385689.

**Results:**

Twenty-four studies with a total of 6208 patients were finally included, including RCTs of 13 drugs. The results of safety showed that there' s no difference in the incidence of SAEs of 13 drugs treated with IPF compared to placebo (*P*>0.05), and it’s also found that Warfarin had a higher all-cause mortality for IPF than placebo (OR = 5.63, 95% CI [1.54 to 20.55]). SUCRA' s scatterplot showed that Pirfenidone, Nintedanib, Sildenafil and Imatinib were lower than placebo, and Warfarin, Ambrisentan and N-acetylcysteine were higher than placebo. The results of effectiveness showed that Nintedanib (MD = -0.08, 95% CI [-0.12 to -0.04]) improved FVC (L)absolute change from baseline in patients better than placebo, and Nintedanib (OR=1.81, 95% CI [1.23 to 2.66]), Pirfenidone (OR=1.85, 95%CI [1.26 to 2.71]) and Pamrevlumab (OR=4.11, 95% CI [1.25 to 13.58]) improved the proportion of patients with a decline in FVC ≥10% predicted better than placebo. SUCRA' s scatterplot showed that Pamrevlumab, Pirfenidone and Nintedanib were lower than placebo, and Warfarin and Ambrisentan were higher than placebo.

**Conclusion:**

Compared with other drugs, Nintedanib and Pirfenidone can significantly slow the decline of lung function in patients with IPF, and the safety is higher. Therefore, they can be further promoted in clinical practice. Warfarin and Ambrisentan shouldn’t be used clinically for IPF as the safety and efficacy of them are poor compared to other drugs and placebo. Pamrevlumab may become important drugs for the treatment of IPF in the future.

**Supplementary Information:**

The online version contains supplementary material available at 10.1186/s12890-024-02861-w.

## Introduction

Idiopathic pulmonary fibrosis (IPF) is a fibrosis interstitial pneumonia of unknown cause. IPF patients usually die within 3-4 years after the diagnosis [1–3]. The 5-year survival rate of IPF patients was 53.7%, with chronic respiratory failure being the leading cause of death in IPF patients and acute exacerbations(AEs) being the second leading cause of death in IPF patients (23.5%) [4]. It is characterized by high morbidity and high mortality [5], and the incidence tends to increase with age [6], among which the proportion of IPF in older males is higher [7]. It can lead to decreased lung function, increased dyspnea and cough, reduced exercise capacity, and deterioration of quality of life as it progresses [8, 9]. Its pathogenesis is closely related to the repair of abnormal alveolar injury [10]. Pirfenidone and Nintedanib are two drugs currently available for the treatment of IPF, both of which were approved by the US Food and Drug Administration in 2014 [11]. In the Phase 3 trial, two drugs slowed the decline in FVC of IPF patients over 1 year compared to placebo [12, 13]. In two other Randomized controlled trials (RCTs) [14, 15], patients with IPF in the Pirfenidone and Nintedanib groups experienced lower AEs than placebo (Pirfenidone:0% vs.Placebo:14.29%; Nintedanib:2.4% vs. Placebo:15.7%). Therefore, these two drugs appear to have good efficacy and safety. However, neither of them had a good effect on symptoms, quality of life, or HRCT of the chest in patients with IPF. In addition, both drugs have gastrointestinal adverse events that can affect long-term treatment adherence in patients [16, 17].

Except Pirfenidone and Nintedanib, some other RCTs of drugs for the treatment of IPF have been reported: In a phase 2 RCT, Pamrevlumab reduced the decline in FVC (% predicted) by 60.3% at week 48, but treatment-induced urgent serious adverse events(SAEs) were observed in 12 (24%) patients in the Pamrevlumab group and 8 (15%) patients in the placebo group [18]; In two RCTs, there was no difference in FVC (% predicted) changes in IPF patients in the Sildenafil group compared with those in the placebo group, and there were no significant differences in AEs (Sildenafil:2/89 vs. Placebo:4/91, *P*=0.68), SAEs (Sildenafil:13/89 vs. Placebo:15/91, *P*=0.73) and all-cause mortality (Sildenafil:2/89 vs. Placebo:4/91, *P*=0.43) [19, 20]; One RCT reported no significant difference between Imatinib and placebo in improving FVC (% predicted) at 96 weeks of follow-up and there were no differences in mortality (Imatinib: 8/59 vs. Placebo:10/60) and AEs (Imatinib:5/59 vs. Placebo:8/60) between the groups [21]; PRM-151 (Recombinant human pentatroxin 2) improved FVC (% predicted) from the baseline to week 28 in patients with IPF in one RCT (difference, +2.3 [90% CI, 1.1 to 3.5], *P* = 0.001), but there was a proportion of SAEs in both groups (Imatinib: 7.8% vs. Placebo: 10.3%) [22]; Results from a phase 2a RCT showed that GLPG1690 improved mean change from baseline in FVC at week 12 (GLPG1690: 25 mL vs. Placebo: -70 mL), and no patients died or had AE-IPFs, but some SAEs occurred in both groups (GLPG1690:1 vs. Placebo:2) [23]; The data from one RCT showed no difference in FVC reduction between the N-acetylcysteine (NAC) 600 mg tid group and the placebo group (60-week change in NAC -0.18 L vs. Placebo -0.19L, *p* = 0.77). In addition, there were no significant differences between NAC and placebo for mortality (6 [4.9%] vs. 3 [2.5%]events, *p*=0.50) or AEs (3 [2.3%] vs. 3 [2.3%] events, *p* > 0.99) [24]; In one RCT with a planned treatment duration of 48 weeks, there was an increase in all-cause mortality in patients with IPF treated with Warfarin (14/72 cases of Warfarin vs. 3/73 cases of Placebo death; *P*=0.005), thus the study was terminated prematurely [25]; One RCT was terminated after enrolling 492 patients (75% of expected enrollment) because the number of patients receiving Ambrisentan may meet pre-specified criteria for disease progression (Ambrisentan: 90 [27.4%] vs. Placebo: 28 [17.2%], patients; *P*=0.01) [26].

These data showed an important problem: the effectiveness of these drugs to treat IPF is different and there are also differences in safety, and it’s difficult to choose more effective and safer drugs among them for the treatment. Therefore, it is necessary to conduct rigorous, objective and systematic quality evaluation of clinical research of different drugs to obtain the safety and efficacy analysis evidence on this basis to guide the clinical use. This study collected all RCTs of IPF reported in literatures, and used systematic review methods to objectively evaluate the safety and efficacy of these drugs for IPF to seek more valuable drugs for the treatment of IPF.

## Methods

This study has been registered in PROSPERO(https://www.crd.york.ac.uk/prospero/), registration number: CRD42023385689. The procedure of this protocol is based on PRISMA-P guidance [27].

### Inclusion criteria

The included studies were all RCTs reported so far for the treatment of IPF with drugs, with or without blinding and allocation concealment, and their language was restricted to English. All studies must meet official diagnostic criteria [11] and the gender, age, race and nationality of participants were not restricted. The experimental group of these RCTs all used drugs independently to treat IPF, and the dose, dosage form and administration method of these drugs were not limited while the control group used placebo matched with the experimental group drugs. The course of treatment of drugs in the test group and the control group was not limited.

### Exclusion criteria

① RCTs with 2 or more drugs for IPF in the experimental group were excluded; ② Literatures with non-RCTs, reviews, case reports, experimental studies, expert experience were excluded; ③ Literatures with duplicate publications and incomplete information were excluded; ④ For repeated publications of the same research results, only the one with the most complete information was retained.

### Outcomes

①Safety outcomes: SAEs and all-cause mortality, and SAEs are defined in the Richeldi L 2014 [13]; ②Effectiveness outcomes: FVC (L) absolute change from baseline, FVC (% predicted)absolute change from baseline and the proportion of patients with decline in FVC≥10% predicted.

### Retrieval strategy

PubMed, EMbase, CENTRAL and MEDLINE were retrieved by computer and the retrieval date was from the construction to November 10, 2022. Theme words and keywords were retrieved combining with literature retrospective and manual retrieval methods, etc. The search terms: “Idiopathic pulmonary fibrosis” OR “Pulmonary fibrosisor” OR “Pulmonary interstitial fibrosis” OR “Interstitial lung disease” OR “IPF”AND“medicine” OR “Drugs” OR “treatment” AND “randomized controlled trial” OR “RCT” OR “Clinical trial”. At the same time, manually retrieve were used to supplement and retrieve relevant documents on the Internet. The search strategy of PubMed is presented in Table S1 in supplemental content.

### Literature screening and data extraction

The literatures were cross-checked by two independent researchers (Wu XZ and Li W) after screening, and those with no unanimous opinion were decided by the 3rd party (Chen YZ). when the literature report is not detailed or the data are insufficient, they try to contact the author by email for details. The design of the data extraction table generally follows the principle of "PICOST" (participants, interventions, comparisons, outcomes, study design, time).

### Quality assessment and risk of bias assessment of literature

The quality criteria of the literature were the modified Jadad scales [28], and risk of bias was recommended by Cochrane Assistance, including: (1) generation of a randomization protocol; (2) concealed grouping; (3) blinding of patients and doctors; (4) blinding of outcome evaluation; (5) incomplete result data; (6) selective results reporting; (7) other biases.

### GRADE evaluates the results

Grading of Recommendations, Assessment, Development and Evaluations (GRADE) [29] was used to evaluate the results of NMA. Refer to the previously published literature [30, 31] for specific methods: For direct comparisons, the estimated starting point of certainty was “high”, and for indirect comparisons, the starting certainty was reduced to “moderate”.

### Data synthesis and analysis

In this study, all network meta-analyses were conducted using a random effects model. Odds ratio (OR) and 95% confidence interval (95% CI) were used for statistical analysis for dichotomous variables and mean difference (MD), and 95% CI were used for continuous variables. *P*<0.05 was statistically significant. When exact mean and SD values were not reported in the included articles, we used the following methods and referred to the previous literature [32]: for Median (IQR) and Median (range), we used online tool (https://www.math.hkbu.edu.hk/~tongt/papers/median2mean.html) for format conversion; for mean (SE) and mean (95% CI), we used the built-in data conversion tool in Revman 5.3 for format conversion. When the included data were sufficiently similar (heterogeneity test: *P*>0.1, I^2^<50%), the NMA can be performed. And consistency models were used simultaneously to evaluate the consistency and inconsistency between data. Due to the inclusion of dual arm studies that directly compare drugs with placebo, only the consistency could be tested rather than the inconsistency.

We ranked the treatment using the surface under the cumulative ranking curve (SUCRA), which is the cumulative relative probability of a treatment being the best option [33, 34]. The higher the rank of SUCRA shows, the higher the level of risk is, for example, a high all-cause mortality value of SUCRA indicates a high all-cause mortality. Influence analysis were performed when there was significant heterogeneity between studies; the funnel plot analysis was used to analyze the publication bias. All the statistical analysis above used Revman 5.3 and Stata 14.0 software.

## Results

### Literature retrieval results

**Fig. 1:**
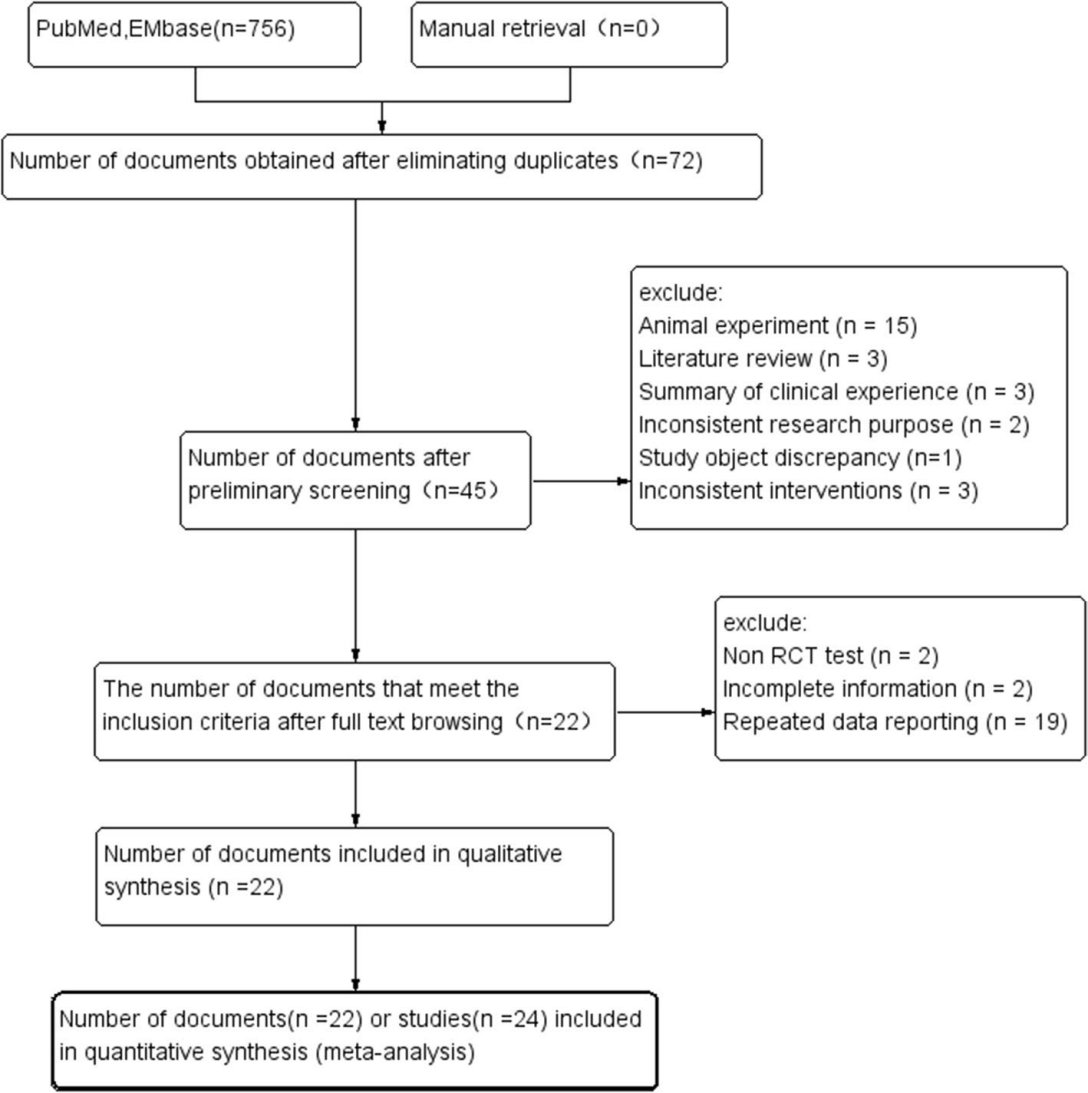
PRISMA literature screening flow chart

PubMed, EMbase, CENTRA Land MEDLINE searched 756 literatures initially, 22 of them, containing 24 studies with a total of 6208 patients, were finally included after layer-by-layer screening, including 3387 in the experimental group and 2821 in the control group and including RCTs of 13 drugs (5 of Nintedanib [13, 14, 35, 36], 4 of Pirfenidone [12, 37, 38], 2 of Sildenafil [19, 20], 1 of Ambrisentan [26], 1 of Pamrevlumab [18], 2 of Bosentan [39, 40], 1 of Macitentan [41], 1 of Imatinib [21], 1 of GLPG1690 [23], 1 of Simtuzumab [42], 1 of Warfarin [25], 2 of PRM-151 [22, 43], 2 of N-acetylcysteine [24, 44]). Figure 1 is a literature screening flowchart developed according to the requirements of the PRISMA statement [27]. The basic characteristics of the included studies were shown in Tables 1 and 2.

**Table 1:** Basic features of the included study (1)

**Studies**	**Industry sponsor**	**Sample(n)**		**Gender (male%)**		**Age(Year)**		**Average course of disease (Year)**		**FVC % predicted**		**Interventions**	
		**E**	**C**	**E**	**C**	**E**	**C**	**E**	**C**	**E**	**C**	**E**	**C**
Daniels CE 2010 [21]	Novartis Pharmaceuticals	59	60	78%	64%	66 (47–79)	67.8 (52–79)	——	——	64.4	65.6	Imatinib 600 mg /day	Placebo
Homma S 2012 [44]	None	38	38	76%	76%	67.6 ± 6.4	68.2 ± 7.7	3.0 ± 3.4	3.2 ± 2.5	89.2 ± 17.8	88.7 ± 15.5	N-acetylcysteine 704.8 mg /day	No treatment (or Placebo)
Jackson RM 2010 [20]	Pfizer UK	14	15	79%	80%	70 ± 12.1	71 ± 6.2	——	——	62.2 ± 16.7	62.7 ± 10.3	Sildenafil 60 mg /day	Placebo
King TE Jr(ASCEND)2014 [12]	Intermune	278	277	79.90%	76.90%	68.4±6.7	67.8±7.3	1.7±1.1	1.7±1.1	67.8±11.2	68.6±10.9	Pirfenidone 2403 mg/day	Placebo
King TE Jr(BUILD-1)2008 [39]	Actelion Pharmaceuticals	71	83	69%	75.9%	65.3±8.4	65.1±9.1	1.2±1.2	1.1±1.0	65.9 ± 10.5	69.5 ± 12.6	Bosentan 250 mg/day	Placebo
King TE Jr(BUILD-3)2011 [40]	Actelion Pharmaceuticals	407	209	72.70%	63.60%	63.8 ± 8.4	63.2 ± 9.1	0.48 (0.05–4.72)	0.50 (0.05–4.72)	74.9 ± 14.8	73.1 ± 15.3	Bosentan 250 mg/day	Placebo
Lancaster L 2020 [35]	None	56	57	80.4%	64.9%	68.8 ±7.6	66.2 ±9.4	1.5 ±1.4	1.5 ±1.4	78.0±17.4	78.1 ± 19.4	Nintedanib 300 mg /day	Placebo
Maher TM(FLORA)2018 [23]	Galapagos	17	6	59%	83%	67·0 (61·0–73·0)	64·0 (54·0–69·0)	1·9 (0·7–3·1)	1·0 (0·5–1·6)	75·3 (67·9–82·7)	69·7 (46·4–92·9)	GLPG1690 600 mg /day	Placebo
Maher TM(INMARK)2019 [36]	Boehringer Ingelheim	116	230	80%	73%	70·5 ±7·7	70·2 ±7·2	0·8 ±0·8	0·9 ±1·0	96·6±15·2	98·0 ± 12·6	Nintedanib 300 mg /day	Placebo
Martinez FJ 2014 [24]	Zambon SpA	133	131	80.50%	74.80%	68.3 ±8.4	67.2 ±8.2	1.0 ±1.0	1.1 ±1.0	72.2 ± 15.9	73.4 ± 14.3	N-acetylcysteine 1800mg /day	Placebo
Noble PW(CAPACITY 004)2011 [37]	Intermune	174	174	68%	74%	65·7 ±8·2	66·3 ±7·5	≤1 Y 48%	≤1 Y: 47%	74·5±14·5	76·2 ± 15·5	Pirfenidone 2403 mg/day	Placebo
Noble PW(CAPACITY 006)2011 [37]	Intermune	171	173	72%	72%	66·8 ±7·9	67·0 ±7·8	≤1 Y: 58%	≤1 Y: 62%	74·9 ±13·2	73·1 ± 14·2	Pirfenidone 2403 mg/day	Placebo
Noth I 2012 [25]	None	72	73	67%	79%	67.3 ± 7.1	66.7 ± 7.4	1.8 ± 1.9	2.1 ± 2.4	58.9 ± 16.2	58.7 ± 16.1	Warfarin (1 mg and 2.5 mg)/day	Placebo
Raghu G 2018 [22]	None	77	39	84%	74%	69.0 ±6.3	67.6 ±7.1	3.7 ±2.2	3.9 ±2.6	67.7±10.9)	67.4 ± 11.4	PRM-151 10 mg/kg/4 weeks	Placebo
Raghu G(ARTEMIS-IPF)2013 [26]	Gilead Sciences	329	163	74.20%	68.10%	65.8 ±7.4	66.1 ±7.1	1.1 ±1.4	0.9 ±1.2	68.7 ± 13.1	69.9 ± 13.8	Ambrisentan 10 mg/day	Placebo
Raghu G(MUSIC)2013 [41]	Actelion Pharmaceuticals	119	59	70.60%	62.70%	65.1±7.85	64.5±6.32	213 (3–1870)D	114 (2–1440)D	76.5 ± 15.6	74.8 ± 14.6	Macitentan 10 mg /day	Placebo
Raghu G(RAINIER)2017 [42]	Gilead Sciences Inc	272	272	84%	83%	67·7 ±7·6	68·5 ±7·1	2·0 ±2·1	2·0 ±2·3	61·4± 12·2	62·3 ± 12·2	Simtuzumab 125 mg/7 days	Placebo
Richeldi L 2020 [18]	FibroGen	50	53	66%	81%	68·3 ±7·1	68·4 ±7·2	1·1 ±1·0	1·5 ±1·2	74·5 ± 11·9	73·1 ± 11·1	Pamrevlumab 30 mg/kg/3 weeks	Placebo
Richeldi L(INPULSIS-1) 2014 [13]	Boehringer Ingelheim	309	204	81.20%	79.90%	66.9±8.4	66.9±8.2	1.7±1.4	1.6±1.4	79.5±17.0	80.5±17.3	Nintedanib 300 mg /day	Placebo
Richeldi L(INPULSIS-2) 2014 [13]	Boehringer Ingelheim	329	219	77.80%	78.10%	66.4±7.9	67.1±7.5	1.6±1.3	1.6±1.3	80.0±18.1	78.1±19.0	Nintedanib 300 mg /day	Placebo
Richeldi L(TOMORROW)2011 [14]	Boehringer Ingelheim	85	85	76.50%	74.10%	65.4±7.8	64.8±8.6	1.0±1.2	1.4±1.5	78.1	77.6	Nintedanib 300 mg /day	Placebo
Taniguchi 2010 [38]	Shionogi & Co., Ltd	108	104	78.70%	77.90%	65.4±6.2	64.7±7.3	≧1 Y: 64.9%	≧1 Y: 60.5%	77.3±16.8	79.1±17.4	Pirfenidone 1800 mg/day	Placebo
van den Blink B 2016 [43]	None	14	6	87%	67%	66.7±7.8	65.5±12.9	——	——	78.8±12.5	63.2±16.7	PRM-151 1, 5 or 10 mg/kg/days 1, 3, 5, 8 and 15	Placebo
Zisman DA(STEP-IPF)2010 [19]	Pfizer	89	91	84%	82%	69.76±8.71	68.20±9.25	2.03±1.94	1.87±1.93	54.89±14.00	58.73±14.12	Sildenafil 60 mg /day	Placebo

*E* Experimental group, *C* Control group, *D* Day, *M* Month, *Y* Year

Data are mean ± SD, mean, or median (IQR) ,or median (Range), or n, unless otherwise stated

**Table 2:** Basic features of the included study (2)

**Studies**	**Interventions**		**Outcomes**	**Course**	**Adverse reactions**
	**Experimental group**	**Control group**			
Daniels CE 2010 [21]	Imatinib 600 mg /day	Placebo	All cause mortality,Serious adverse events,FVC(L)	96W	Described
Homma S 2012 [44]	N-acetylcysteine 704.8 mg /day	No treatment (or Placebo)	FVC(L)	48W	Described
Jackson RM 2010 [20]	Sildenafil 60 mg /day	Placebo	FVC (%)	6M	Described
King TE Jr(ASCEND)2014 [12]	Pirfenidone 2403 mg/day	Placebo	All cause mortality,Serious adverse events,FVC≥10%	52W	Described
King TE Jr(BUILD-1)2008 [39]	Bosentan 250 mg/day	Placebo	All cause mortality,Serious adverse events	12M	Described
King TE Jr(BUILD-3)2011 [40]	Bosentan 250 mg/day	Placebo	All cause mortality,Serious adverse events,FVC(L)	1Y	Described
Lancaster L 2020 [35]	Nintedanib 300 mg /day	Placebo	All cause mortality,Serious adverse events,FVC(L),FVC (%),FVC≥10%	6M	Described
Maher TM(FLORA)2018 [23]	GLPG1690 600 mg /day	Placebo	Serious adverse events,FVC(L)	12W	Described
Maher TM(INMARK)2019 [36]	Nintedanib 300 mg /day	Placebo	Serious adverse events,FVC(L)	12W	Described
Martinez FJ 2014 [24]	N-acetylcysteine 1800mg /day	Placebo	All cause mortality,Serious adverse events	60W	Described
Noble PW(CAPACITY 004)2011 [37]	Pirfenidone 2403 mg/day	Placebo	All cause mortality,Serious adverse events,FVC (%),FVC≥10%	72W	Described
Noble PW(CAPACITY 006)2011 [37]	Pirfenidone 2403 mg/day	Placebo	All cause mortality,Serious adverse events,FVC (%),FVC≥10%	72W	Described
Noth I 2012 [25]	Warfarin (1 mg and 2.5 mg)/day	Placebo	All cause mortality,Serious adverse events,FVC(L),FVC (%),FVC≥10%	48W	Described
Raghu G 2018 [22]	PRM-151 10 mg/kg/4 weeks	Placebo	Serious adverse events	28W	Described
Raghu G(ARTEMIS-IPF)2013 [26]	Ambrisentan 10 mg/day	Placebo	All cause mortality,Serious adverse events,FVC (%),FVC≥10%	84W	Described
Raghu G(MUSIC)2013 [41]	Macitentan 10 mg /day	Placebo	All cause mortality,Serious adverse events,FVC(L)	12M	Described
Raghu G(RAINIER)2017 [42]	Simtuzumab 125 mg/7 days	Placebo	All cause mortality,Serious adverse events	82W	Described
Richeldi L 2020 [18]	Pamrevlumab 30 mg/kg/3 weeks	Placebo	All cause mortality,Serious adverse events,FVC(L),FVC (%),FVC≥10%	48W	Described
Richeldi L(INPULSIS-1) 2014 [13]	Nintedanib 300 mg /day	Placebo	All cause mortality,Serious adverse events,FVC≥10%	52W	Described
Richeldi L(INPULSIS-2) 2014 [13]	Nintedanib 300 mg /day	Placebo	All cause mortality,Serious adverse events,FVC≥10%	52W	Described
Richeldi L(TOMORROW)2011 [14]	Nintedanib 300 mg /day	Placebo	All cause mortality,Serious adverse events,FVC(L),FVC (%)	52W	Described
Taniguchi 2010 [38]	Pirfenidone 1800 mg/day	Placebo	All cause mortality	52W	Described
van den Blink B 2016 [43]	PRM-151 1, 5 or 10 mg/kg/days 1, 3, 5, 8 and 15	Placebo	FVC(L),FVC (%)	57D	Described
Zisman DA(STEP-IPF)2010 [19]	Sildenafil 60 mg /day	Placebo	All cause mortality,Serious adverse events,FVC (%)	12W	Described

*E* Experimental group, *C* Control group, *W* Week, *D* Day, *M* Month, *Y* Year, *FVC* Forced vital capacity

FVC (L): FVC (L) absolute change from baseline;

FVC (%): FVC (% predicted)absolute change from baseline;

FVC≥10%: The proportion of patients with decline in FVC≥10% predicted

### Quality assessment

The 24 included studies were RCTs, all of which mentioned the use of a randomization method and described the comparability of baseline data between the two groups, and there were no incomplete data being reported or data missing. All of them also described the treatment and outcome measures in the experimental and control groups, and 22 studies described specific allocation concealment methods and blinding. The modified Jadad scale [28] was used to evaluate the 24 included studies, of which 19 studies were with 7 points, 3 studies were with 6 points, 1 study was with 5 points, and 1 study was with 3 points. There were 23 high-quality studies and 1 low-quality study. The results of the quality evaluation were shown in Table S2 in supplemental content.

### Cochrane risk of bias assessment results

**Fig. 2:**
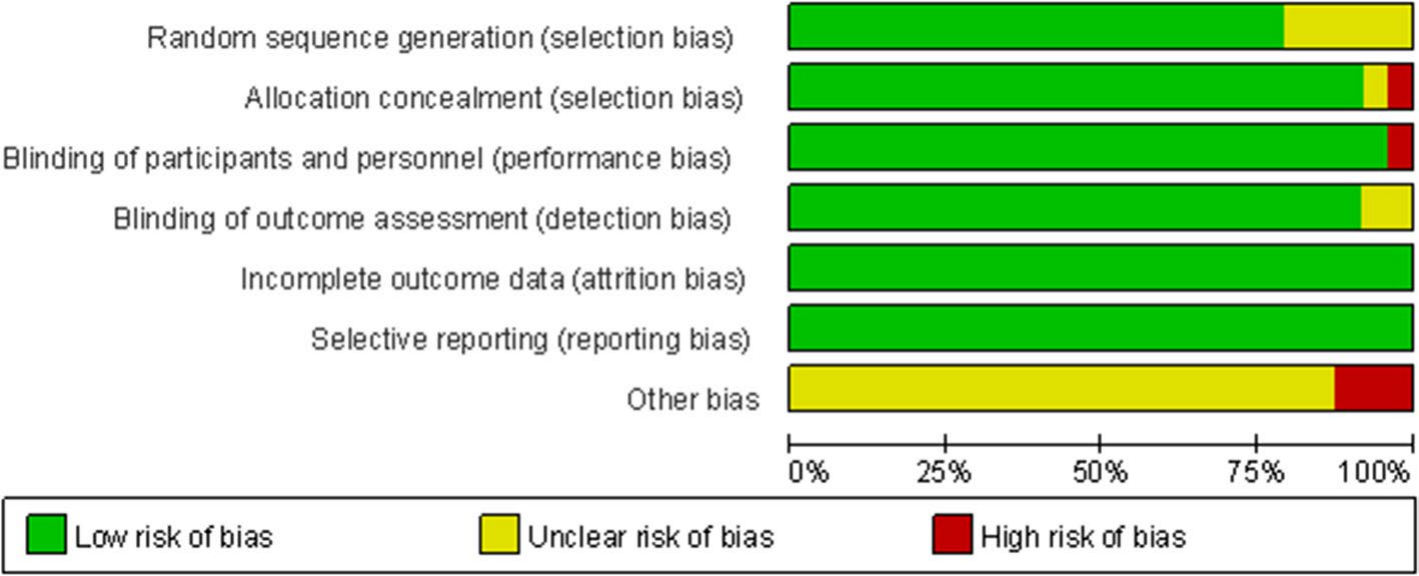
Bias risk percentage

**Fig. 3:**
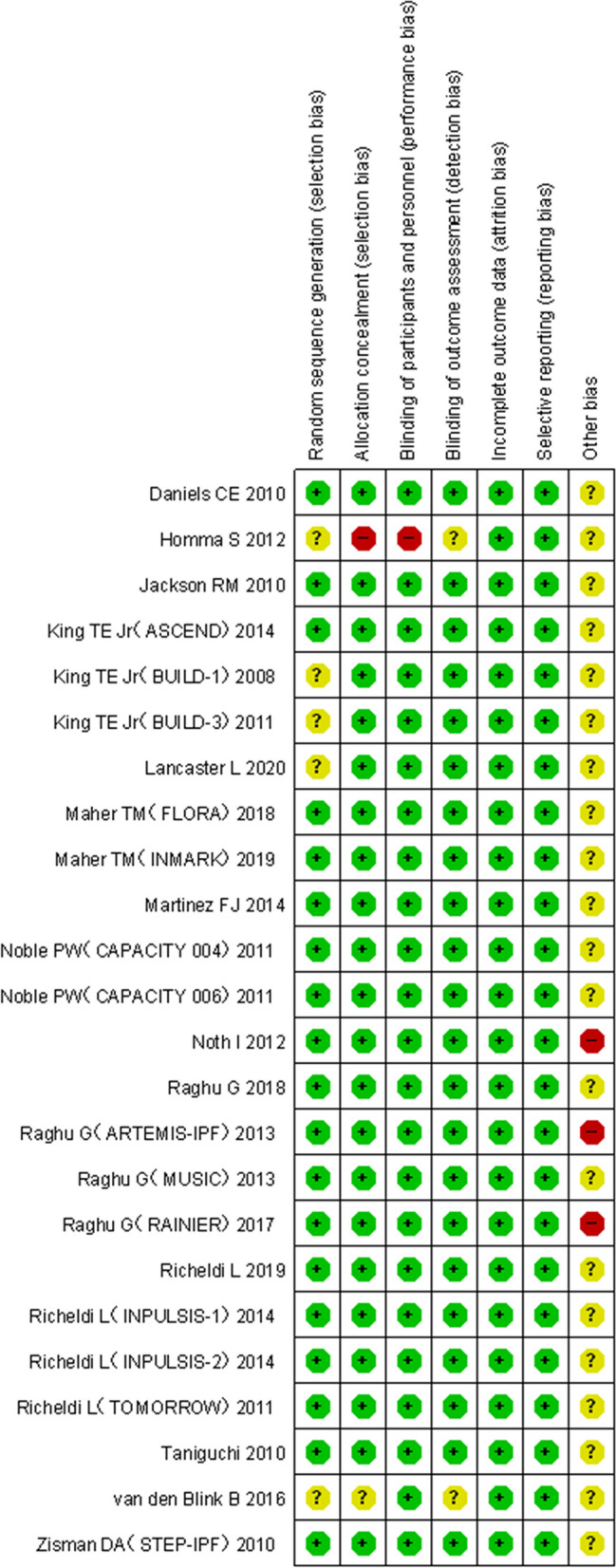
Bias risk summary chart

The results showed that the low risk proportion of random sequence generation in the selection bias of the 24 studies was about 79.76%, the moderate risk was about 17.26%, and the high risk was about 2.98% (Fig. 2), so the included studies had less selection, implementation and measurement bias, and the bias statistics of each study were shown in Fig. 3.

### Security analysis

#### SAEs

**Fig. 4:**
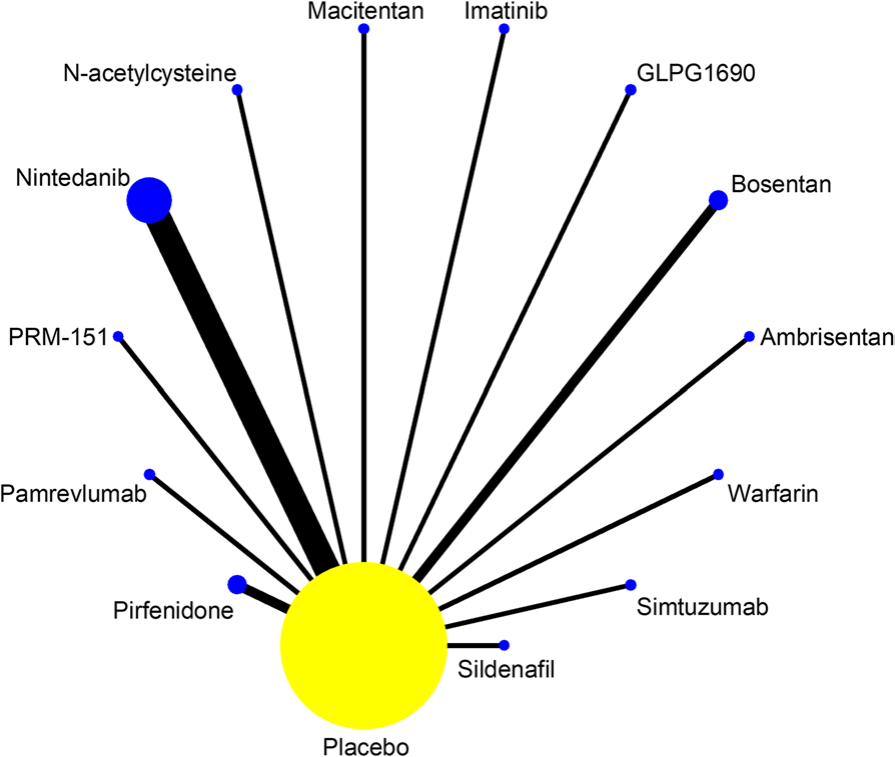
Network evidence map of SAEs. A total of 19 studies reported SAEs in the treatment of IPF with 13 drugs: 1 of Ambrisentan, 2 of Bosentan, 1 of GLPG1690, 1 of Imatinib, 1 of Macitentan, 1 of N-acetylcysteine, 5 of Nintedanib, 1 of Pamrevlumab, 2 of Pirfenidone, 1 of PRM-151, 1 of Sildenafil, 1 of Simtuzumab, 1 of Warfarin

Of the 24 studies included, a total of 19 studies reported SAEs in the treatment of IPF with 13 drugs, as shown in Fig. 4. Statistical analyses were performed with OR and 95%CI as the effect size, and the results of heterogeneity test showed I^2^=0%, *P*=0.623, thus it met the criteria of *P*>0.1, I^2^<50%, and the effect sizes could be combined for meta-analysis. The consistency model results showed that the effect sizes (Log OR) of all study were approximately between 0 and 2, indicating that the consistency of the results was credible, as shown in Figure S1 in supplemental content. The results of the network meta-analysis (NMA) showed (Table 3, Figure S2 in supplemental content) that there was no difference in the incidence of SAEs between the 13 drugs and placebo (*P*>0.05). Comparisons between drugs showed that Warfarin had a higher incidence of SAEs than Bosentan (OR = 2.49, 95% CI [1.06 to 5.89], low certainty of evidence) and GLPG1690 (OR = 16.75, 95% CI [1.06 to 263.92], low certainty of evidence); Ambrisentan had a higher incidence of SAEs than Bosentan (OR=1.88, 95% CI [1.04 to 3.38], low certainty of evidence). The SUCRA ranking showed: Warfarin (89.4) > Ambrisentan(81.6) > Pamrevlumab(80.1) > N-acetylcysteine(66.1) > Simtuzumab(54.1) > Pirfenidone(48.5) > Placebo(48) > Imatinib(44) > Nintedanib(42.7) > Sildenafil(38.7) > Macitentan(37.6) > PRM151(34) > Bosentan(29) > GLPG1690(6.1). Higher values of SUCRA indicate higher incidence of SAEs. As shown in Table 4 and Figure S3 in supplemental content.

**Table 3:** Results of network meta-analysis of the incidence of SAEs

Warfarin													
1.33 (0.52,3.42) ⊕⊕⊝⊝low2,4	Ambrisentan												
1.18 (0.33,4.22) ⊕⊕⊝⊝low2,4	0.89 (0.29,2.69) ⊕⊕⊝⊝low2,4	Pamrevlumab											
1.63 (0.58,4.56) ⊕⊕⊝⊝low2,4	1.23 (0.54,2.77) ⊕⊕⊝⊝low2,4	1.38 (0.42,4.52) ⊕⊕⊕⊝moderate4	N-acetylcysteine										
1.96 (0.82,4.71) ⊕⊕⊝⊝low2,4	1.48 (0.80,2.72) ⊕⊕⊝⊝low2,4	1.67 (0.58,4.78) ⊕⊕⊝⊝low2,4	1.21 (0.58,2.51) ⊕⊕⊝⊝low2,4	Simtuzumab									
2.09 (0.90,4.83) ⊕⊕⊝⊝low2,4	1.57 (0.90,2.75) ⊕⊕⊝⊝low2,4	1.77 (0.63,4.93) ⊕⊕⊕⊝moderate4	1.28 (0.64,2.56) ⊕⊕⊕⊝moderate4	1.06 (0.69,1.64) ⊕⊕⊝⊝low2,4	Pirfenidone								
2.09 (0.94,4.66) ⊕⊕⊕⊝moderate2	1.57 (0.96,2.59) ⊕⊕⊕⊝moderate2	1.78 (0.66,4.80) ⊕⊕⊕⊕high	1.28 (0.67,2.45) ⊕⊕⊕⊕high	1.07 (0.75,1.51) ⊕⊕⊕⊝moderate2	1.00 (0.78,1.29) ⊕⊕⊕⊕high	Placebo							
2.21 (0.72,6.74) ⊕⊕⊝⊝low2,4	1.66 (0.66,4.18) ⊕⊕⊝⊝low2,4	1.87 (0.53,6.62) ⊕⊕⊕⊝moderate4	1.36 (0.49,3.72) ⊕⊕⊕⊝moderate4	1.12 (0.48,2.64) ⊕⊕⊝⊝low2,4	1.06 (0.47,2.40) ⊕⊕⊕⊝moderate4	1.06 (0.49,2.29) ⊕⊕⊕⊕high	Imatinib						
2.18 (0.94,5.01) ⊕⊕⊝⊝low2,4	1.64 (0.94,2.84) ⊕⊕⊝⊝low2,4	1.85 (0.67,5.12) ⊕⊕⊕⊝moderate4	1.34 (0.67,2.65) ⊕⊕⊕⊝moderate4	1.11 (0.73,1.69) ⊕⊕⊝⊝low2,4	1.04 (0.74,1.47) ⊕⊕⊕⊝moderate4	1.04 (0.82,1.31) ⊕⊕⊕⊕high	0.98 (0.44,2.22) ⊕⊕⊕⊝moderate4	Nintedanib					
2.42 (0.77,7.53) ⊕⊕⊝⊝low2,4	1.82 (0.70,4.69) ⊕⊕⊝⊝low2,4	2.05 (0.57,7.37) ⊕⊕⊕⊝moderate4	1.48 (0.53,4.17) ⊕⊕⊕⊝moderate4	1.23 (0.51,2.96) ⊕⊕⊝⊝low2,4	1.16 (0.50,2.70) ⊕⊕⊕⊝moderate4	1.15 (0.51,2.59) ⊕⊕⊕⊕high	1.09 (0.36,3.35) ⊕⊕⊕⊝moderate4	1.11 (0.48,2.58) ⊕⊕⊕⊝moderate4	Sildenafil				
2.38 (0.84,6.73) ⊕⊕⊝⊝low2,4	1.79 (0.78,4.11) ⊕⊕⊝⊝low2,4	2.02 (0.61,6.67) ⊕⊕⊕⊝moderate4	1.46 (0.58,3.69) ⊕⊕⊕⊝moderate4	1.21 (0.57,2.57) ⊕⊕⊝⊝low2,4	1.14 (0.56,2.32) ⊕⊕⊕⊝moderate4	1.14 (0.58,2.21) ⊕⊕⊕⊕high	1.08 (0.39,2.99) ⊕⊕⊕⊝moderate4	1.09 (0.54,2.21) ⊕⊕⊕⊝moderate4	0.98 (0.35,2.80) ⊕⊕⊕⊝moderate4	Macitentan			
2.83 (0.60,13.35) ⊕⊕⊝⊝low2,4	2.13 (0.52,8.80) ⊕⊕⊝⊝low2,4	2.40 (0.46,12.62) ⊕⊕⊕⊝moderate4	1.74 (0.40,7.61) ⊕⊕⊕⊝moderate4	1.44 (0.36,5.69) ⊕⊕⊝⊝low2,4	1.36 (0.35,5.25) ⊕⊕⊕⊝moderate4	1.35 (0.36,5.10) ⊕⊕⊕⊕high	1.28 (0.28,5.97) ⊕⊕⊕⊝moderate4	1.30 (0.34,5.01) ⊕⊕⊕⊝moderate4	1.17 (0.25,5.55) ⊕⊕⊕⊝moderate4	1.19 (0.27,5.25) ⊕⊕⊕⊝moderate4	PRM151		
**2.49 (1.06,5.89)** ⊕⊕⊝⊝low2,4	**1.88 (1.04,3.38)** ⊕⊕⊝⊝low2,4	2.12 (0.75,6.00) ⊕⊕⊕⊝moderate4	1.53 (0.75,3.13) ⊕⊕⊕⊝moderate4	1.27 (0.79,2.03) ⊕⊕⊝⊝low2,4	1.20 (0.80,1.79) ⊕⊕⊕⊝moderate4	1.19 (0.87,1.63) ⊕⊕⊕⊕high	1.13 (0.49,2.61) ⊕⊕⊕⊝moderate4	1.15 (0.78,1.69) ⊕⊕⊕⊝moderate4	1.03 (0.43,2.46) ⊕⊕⊕⊝moderate4	1.05 (0.50,2.18) ⊕⊕⊕⊝moderate4	0.88 (0.23,3.45) ⊕⊕⊕⊝moderate4	Bosentan	
**16.75 (1.06,263.92)** ⊕⊕⊝⊝low2,4	12.59 (0.86,184.67) ⊕⊕⊝⊝low2,4	14.21 (0.85,238.28) ⊕⊕⊕⊝moderate4	10.28 (0.68,155.45) ⊕⊕⊕⊝moderate4	8.52 (0.60,122.08) ⊕⊕⊝⊝low2,4	8.03 (0.57,113.76) ⊕⊕⊕⊝moderate4	8.00 (0.57,111.96) ⊕⊕⊕⊕high	7.58 (0.48,118.62) ⊕⊕⊕⊝moderate4	7.70 (0.54,108.87) ⊕⊕⊕⊝moderate4	6.93 (0.44,109.50) ⊕⊕⊕⊝moderate4	7.04 (0.46,106.96) ⊕⊕⊕⊝moderate4	5.92 (0.31,113.49) ⊕⊕⊕⊝moderate4	6.71 (0.47,95.69) ⊕⊕⊕⊝moderate4	GLPG1690

Data are OR(95%Cl)

1:Certainty lowered for imprecision

2:Certainty lowered for individual study risk of bias

3:Certainty lowered two levels for imprecision

4:Certainty lowered for indirectness

GRADE Working Group grades of evidence – High quality: Further research is very unlikely to change our confidence in the estimate of effect; Moderate quality: Further research is likely to have an important impact on our confidence in the estimate of effect and may change the estimate; Low quality: Further research is very likely to have an important impact on our confidence in the estimate of effect and is likely to change the estimate; Very low quality: We are very uncertain about the estimate

**Table 4:** SUCRA ranking of the incidence of SAEs

**Treatment**	**SUCRA**	**PrBest**	**MeanRank**
Warfarin	89.4	44.9	2.4
Ambrisentan	81.6	10.3	3.4
Pamrevlumab	80.1	30.5	3.6
N-acetylcysteine	66.1	5.3	5.4
Simtuzumab	54.1	0.1	7
Pirfenidone	48.5	0	7.7
Placebo	48	0	7.8
Imatinib	44	1.6	8.3
Nintedanib	42.7	0	8.5
Sildenafil	38.7	1.2	9
Macitentan	37.6	0.7	9.1
PRM151	34	4.4	9.6
Bosentan	29	0	10.2
GLPG1690	6.1	1	13.2

Higher values of SUCRA indicate higher incidence of SAEs

#### All-cause mortality

**Fig. 5:**
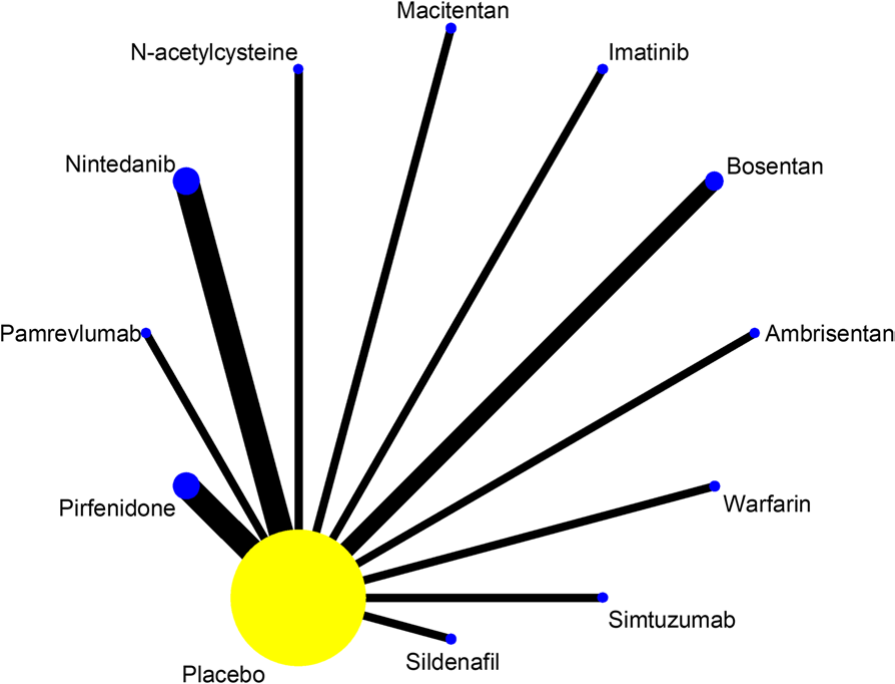
Network evidence map of all-cause mortality. A total of 16 studies reported the all-cause mortality of IPF treated with 11 drugs: 1 of Ambrisentan, 2 of Bosentan,1 of Imatinib, 1 of Macitentan, 1 of N-acetylcysteine, 3 of Nintedanib, 1 of Pamrevlumab, 3 of Pirfenidone, 1 of Sildenafil, 1 of Simtuzumab, 1 of Warfarin

Of the 24 studies included, 16 reported the all-cause mortality of IPF treated with 11 drugs, as shown in Fig. 5. The results of heterogeneity tests showed I^2^=28.7% and *P*=0.136, which could be combined for meta-analysis. The consistency model results showed that all study effect sizes (Log OR) were approximately between 0 and 2, indicating that the consistency of the results was credible, as shown in Figure S4 in supplemental content. The results of the NMA (Table 5, Figure S5 in supplemental content) showed that Warfarin had higher all-cause mortality than placebo (OR = 5.63, 95% CI [1.54 to 20.55], moderate certainty of evidence), and there was no difference in all-cause mortality compared with placebo for the remaining 10 drugs (*P*>0.05). Comparisons between drugs showed that Warfarin had higher all-cause mortality than Bosentan (OR = 4, 73, 95% CI [1.17 to 19.06], low certainty of evidence), Simtuzumab (OR = 5.84, 95% CI [1.44 to 23.61], low certainty of evidence), Imatinib (OR=7.18, 95% CI[1.39,37.05], low certainty of evidence), Pirfenidone (OR=8.17, 95%CI[2.10,31.82], low certainty of evidence), Nintedanib (OR=8.38, 95%CI[2.14,32.84], low certainty of evidence), Sildenafil (OR=11.26, 95% CI [1.31 to 97.22], low certainty of evidence) and Pamrevlumab (OR=11.26, 95% CI [1.62 to 78.32], low certainty of evidence); Ambrisentan had higher all-cause mortality than Pirfenidone (OR = 3.26, 95% CI [1.20 to 8.85], low certainty of evidence) and Nintedanib (OR = 3.34, 95% CI [1.22 to 9.15], low certainty of evidence). The SUCRA ranking showed: Warfarin (96.6) > Ambrisentan(82.9) > N-acetylcysteine(75) > Bosentan(60.9) > Macitentan(54.1) > Placebo(51.3) > Simtuzumab(48.2) > Imatinib(36.4) > Pirfenidone(25.6) > Nintedanib(24.2) > Sildenafil(23.7) > Pamrevlumab(21.2). Higher values of SUCRA indicate higher all-cause mortality. As shown in Table 6, Figure S6 in supplemental content.

**Table 5:** Results of network meta-analysis of all-cause mortality

Warfarin											
2.51 (0.52,12.20) ⊕⊕⊝⊝low2,4	Ambrisentan										
2.79 (0.41,18.91) ⊕⊕⊝⊝low2,4	1.11 (0.21,5.95) ⊕⊕⊝⊝low2,4	N-acetylcysteine									
**4.73 (1.17,19.06)** ⊕⊕⊝⊝low2,4	1.89 (0.66,5.36) ⊕⊕⊝⊝low2,4	1.69 (0.38,7.58) ⊕⊕⊕⊝moderate4	Bosentan								
5.01 (0.84,29.68) ⊕⊕⊝⊝low2,4	2.00 (0.44,9.15) ⊕⊕⊝⊝low2,4	1.79 (0.28,11.55) ⊕⊕⊕⊝moderate4	1.06 (0.28,3.98) ⊕⊕⊕⊝moderate4	Macitentan							
**5.63 (1.54,20.55)** ⊕⊕⊕⊝moderate2	2.25 (0.91,5.57) ⊕⊕⊕⊝moderate2	2.02 (0.49,8.24) ⊕⊕⊕⊕high	1.19 (0.71,1.99) ⊕⊕⊕⊕high	1.13 (0.33,3.82) ⊕⊕⊕⊕high	Placebo						
**5.84 (1.44,23.61)** ⊕⊕⊝⊝low2,4	2.33 (0.82,6.65) ⊕⊕⊝⊝low2,4	2.09 (0.47,9.39) ⊕⊕⊝⊝low2,4	1.23 (0.59,2.58⊕⊕⊝⊝low2,4	1.17 (0.31,4.41) ⊕⊕⊝⊝low2,4	1.04 (0.61,1.75) ⊕⊕⊕⊝moderate2	Simtuzumab					
**7.18 (1.39,37.05)** ⊕⊕⊝⊝low2,4	2.86 (0.74,11.12) ⊕⊕⊝⊝low2,4	2.57 (0.46,14.52) ⊕⊕⊕⊝moderate4	1.52 (0.49,4.71) ⊕⊕⊕⊝moderate4	1.43 (0.29,6.99) ⊕⊕⊕⊝moderate4	1.27 (0.47,3.49) ⊕⊕⊕⊕high	1.23 (0.39,3.83) ⊕⊕⊝⊝low2,4	Imatinib				
**8.17 (2.10,31.82)** ⊕⊕⊝⊝low2,4	**3.26 (1.20,8.85)** ⊕⊕⊝⊝low2,4	2.92 (0.67,12.69) ⊕⊕⊕⊝moderate4	1.73 (0.89,3.35) ⊕⊕⊕⊝moderate4	1.63 (0.45,5.93) ⊕⊕⊕⊝moderate4	1.45 (0.96,2.20) ⊕⊕⊕⊕high	1.40 (0.72,2.74) ⊕⊕⊝⊝low2,4	1.14 (0.38,3.39) ⊕⊕⊕⊝moderate4	Pirfenidone			
**8.38 (2.14,32.84)** ⊕⊕⊝⊝low2,4	**3.34 (1.22,9.15)** ⊕⊕⊝⊝low2,4	3.00 (0.69,13.09) ⊕⊕⊕⊝moderate4	1.77 (0.90,3.48) ⊕⊕⊕⊝moderate4	1.67 (0.46,6.12) ⊕⊕⊕⊝moderate4	1.49 (0.96,2.30) ⊕⊕⊕⊕high	1.44 (0.73,2.84) ⊕⊕⊝⊝low2,4	1.17 (0.39,3.50) ⊕⊕⊕⊝moderate4	1.03 (0.56,1.88) ⊕⊕⊕⊝moderate4	Nintedanib		
**11.26 (1.31,97.22)** ⊕⊕⊝⊝low2,4	4.49 (0.64,31.50) ⊕⊕⊝⊝low2,4	4.03 (0.44,37.30) ⊕⊕⊕⊝moderate4	2.38 (0.39,14.39) ⊕⊕⊕⊝moderate4	2.25 (0.27,18.60) ⊕⊕⊕⊝moderate4	2.00 (0.36,11.20) ⊕⊕⊕⊕high	1.93 (0.32,11.69) ⊕⊕⊝⊝low2,4	1.57 (0.21,11.55) ⊕⊕⊕⊝moderate4	1.38 (0.23,8.12) ⊕⊕⊕⊝moderate4	1.34 (0.23,7.95) ⊕⊕⊕⊝moderate4	Sildenafil	
**11.26 (1.62,78.32)** ⊕⊕⊝⊝low2,4	4.49 (0.82,24.72) ⊕⊕⊝⊝low2,4	4.03 (0.54,30.28) ⊕⊕⊕⊝moderate4	2.38 (0.51,11.03) ⊕⊕⊕⊝moderate4	2.25 (0.34,14.91) ⊕⊕⊕⊝moderate4	2.00 (0.47,8.47) ⊕⊕⊕⊕high	1.93 (0.42,8.97) ⊕⊕⊝⊝low2,4	1.57 (0.27,9.13) ⊕⊕⊕⊝moderate4	1.38 (0.31,6.20) ⊕⊕⊕⊝moderate4	1.34 (0.30,6.07) ⊕⊕⊕⊝moderate4	1.00 (0.11,9.47) ⊕⊕⊕⊝moderate4	Pamrevlumab

Data are OR(95%Cl)

1:Certainty lowered for imprecision

2:Certainty lowered for individual study risk of bias

3:Certainty lowered two levels for imprecision

4:Certainty lowered for indirectness

GRADE Working Group grades of evidence – High quality: Further research is very unlikely to change our confidence in the estimate of effect; Moderate quality: Further research is likely to have an important impact on our confidence in the estimate of effect and may change the estimate; Low quality: Further research is very likely to have an important impact on our confidence in the estimate of effect and is likely to change the estimate; Very low quality: We are very uncertain about the estimate

**Table 6:** SUCRA ranking of all-cause mortality

**Treatment**	**SUCRA**	**PrBest**	**MeanRank**
Warfarin	96.6	75.7	1.4
Ambrisentan	82.9	9.4	2.9
N-acetylcysteine	75	11.9	3.8
Bosentan	60.9	0.1	5.3
Macitentan	54.1	2	6
Placebo	51.3	0	6.4
Simtuzumab	48.2	0	6.7
Imatinib	36.4	0.2	8
Pirfenidone	25.6	0	9.2
Nintedanib	24.2	0	9.3
Sildenafil	23.7	0.6	9.4
Pamrevlumab	21.2	0.2	9.7

Higher values of SUCRA indicate higher all-cause mortality

**Fig. 6:**
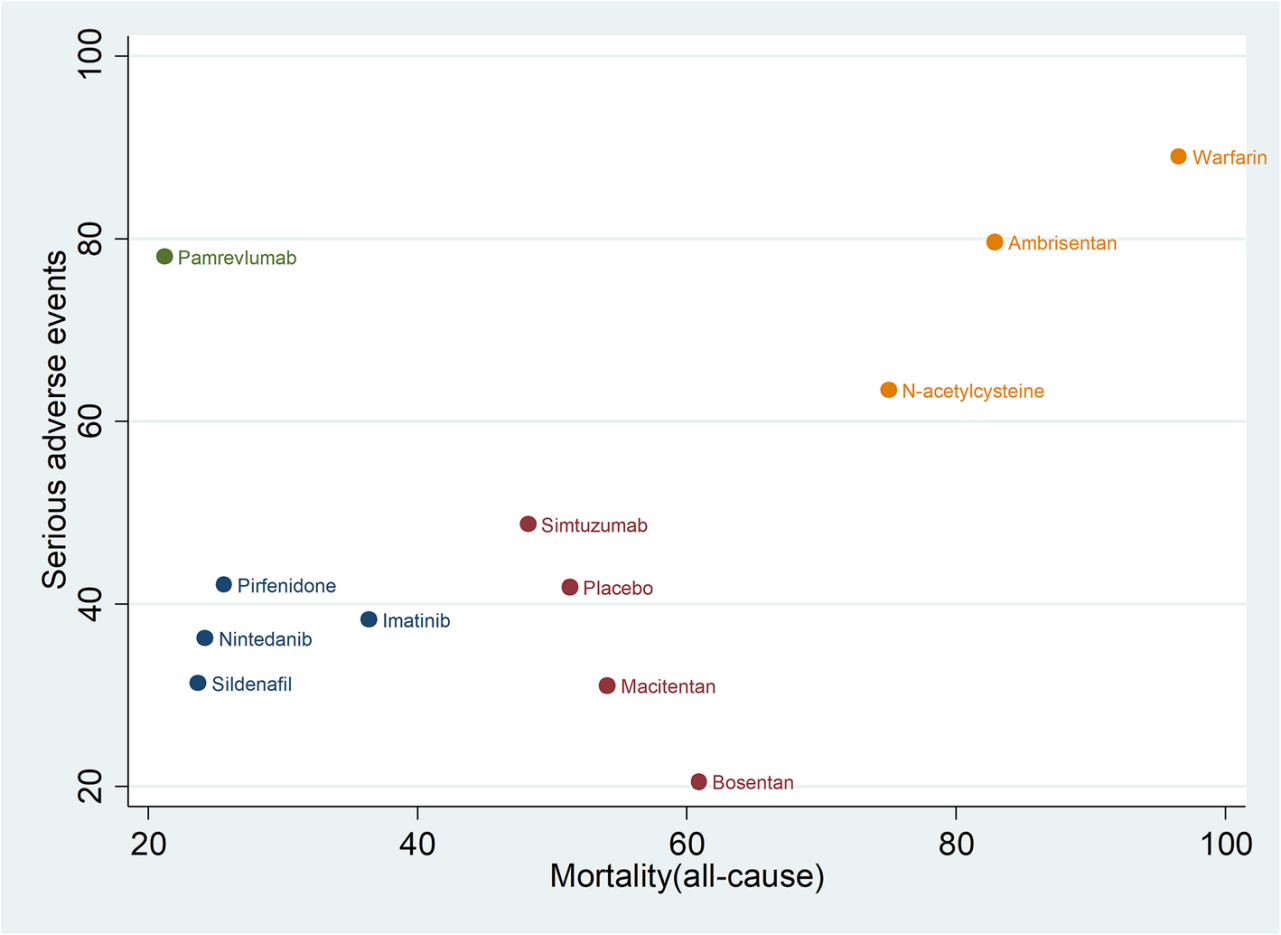
The scatterplot combining the results of the incidence of SAEs and all-cause mortality (SUCRA values). The horizontal coordinate represents SUCRA values for all-cause mortality and the vertical coordinate represents SUCRA values for SAEs.If drugs are positioned further to the upper right of the graph, it means that the higher their SUCRA values, the higher their risk; if drugs are positioned further to the lower left of the graph, it means that the lower their SUCRA values are lower, the lower the risk

Combining the results from SAEs and all-cause mortality, the scatterplot showed (Fig. 6): the SUCRA values of Pirfenidone, Nintedanib, Sildenafil and Imatinib were lower than those of placebo, the SUCRA values of Simtuzumab, Macitentan and Bosentan were approximately equal to those of placebo, and the SUCRA values of Warfarin, Ambrisentan and N-acetylcysteine were higher than those of placebo. In addition, the SUCRA value of all-cause mortality of Pamrevlumab was lower than placebo, but the SUCRA value of the incidence of SAEs was higher than placebo.

### Effectiveness analysis

#### FVC (L) absolute change from baseline

**Fig. 7:**
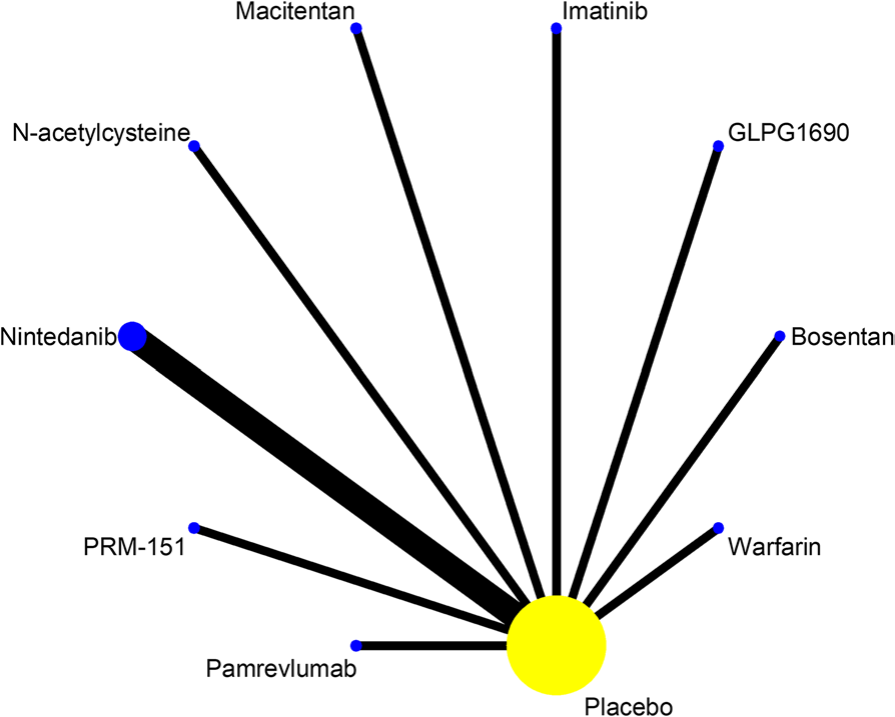
Network evidence map of FVC (L) absolute change from baseline. A total of 11 studies reported the FVC (% predicted) absolute change from baseline in patients with IPF treated with 9 drugs:1 of Bosentan, 1 of GLPG1690, 1 of Imatinib, 1 of Macitentan, 1 of N-acetylcysteine, 3 of Nintedanib, 1 of Pamrevlumab, 1 of PRM-151, 1 of Warfarin

A total of 11 of the 24 included studies reported on 9 drugs to treat FVC (L) absolute change from baseline in patients with IPF, as shown in Fig. 7. Statistical analyses were performed by using MD and 95%CI as effect sizes. The results of the heterogeneity test showed I^2^=6.5% and P=0.382, which could be combined for meta-analysis. The consistency model results showed that all study effect sizes (MD) were approximately between -2 and 0, indicating that the consistency of the results is credible, as shown in Figure S7 in supplemental content. The results of the NMA (Table 7, Figure S8 in supplemental content) showed that the improvement of FVC (L) absolute change from baseline by Nintedanib (MD=-0.08, 95% CI [-0.12 to -0.04], high certainty of evidence) and PRM151 (MD=-0.13, 95% CI [-0.25 to -0.01], moderate certainty of evidence) was better than that by placebo and there was no difference in improvement with placebo for the remaining 7 drugs (*P*>0.05). The results of the comparison between drugs showed: Nintedanib (MD=-0.12, 95% CI [-0.22 to -0.02], low certainty of evidence) and PRM151 (MD=-0.17, 95% CI [-0.32 to -0.01], very low certainty of evidence) improved FVC (L) absolute change from baseline better than Warfarin. The SUCRA ranking showed: Warfarin (85.2) > Imatinib (73.2) > Placebo(72.6) > Macitentan(61.3) > Bosentan(54.8) > N-acetylcysteine(47.1) > Nintedanib(36.2) > GLPG1690(34) > PRM151(22.7) > Pamrevlumab(13). Higher values of SUCRA indicate higher values of decline in FVC (L) absolute change from baseline. As shown in Table 8 and Figure S9 in supplemental content.

**Table 7:** Results of network meta-analysis of FVC (L) absolute change from baseline

Warfarin									
-0.03 (-0.18,0.12) ⊕⊕⊝⊝low2,4	Imatinib								
-0.04 (-0.14,0.06) ⊕⊕⊕⊝moderate2	-0.01 (-0.13,0.11) ⊕⊕⊕⊕high	Placebo							
-0.04 (-0.37,0.29) ⊕⊕⊝⊝low2,4	-0.01 (-0.35,0.33) ⊕⊕⊕⊝moderate4	-0.00 (-0.32,0.32) ⊕⊕⊕⊕high	Macitentan						
-0.08 (-0.26,0.10) ⊕⊕⊝⊝low2,4	-0.05 (-0.24,0.14) ⊕⊕⊕⊝moderate4	-0.04 (-0.19,0.11) ⊕⊕⊕⊕high	-0.04 (-0.39,0.31) ⊕⊕⊕⊝moderate4	Bosentan					
-0.10 (-0.25,0.05) ⊕⊝⊝⊝ very low1,2,4	-0.07 (-0.24,0.10) ⊕⊝⊝⊝ very low1,2,4	-0.06 (-0.17,0.05) ⊕⊕⊝⊝ low1,2	-0.06 (-0.40,0.28) ⊕⊝⊝⊝ very low1,2,4	-0.02 (-0.21,0.17) ⊕⊝⊝⊝ very low1,2,4	N-acetylcysteine				
**-0.12 (-0.22,-0.02)** ⊕⊕⊝⊝low2,4	-0.09 (-0.22,0.03) ⊕⊕⊕⊝moderate4	**-0.08 (-0.12,-0.04)** ⊕⊕⊕⊕high	-0.08 (-0.40,0.24) ⊕⊕⊕⊝moderate4	-0.04 (-0.19,0.11) ⊕⊕⊕⊝moderate4	-0.02 (-0.14,0.10) ⊕⊝⊝⊝ very low1,2,4	Nintedanib			
-0.14 (-0.31,0.04) ⊕⊕⊝⊝low2,4	-0.10 (-0.29,0.08) ⊕⊕⊕⊝moderate4	-0.09 (-0.24,0.05) ⊕⊕⊕⊕high	-0.09 (-0.44,0.25) ⊕⊕⊕⊝moderate4	-0.05 (-0.26,0.15) ⊕⊕⊕⊝moderate4	-0.04 (-0.22,0.15) ⊕⊝⊝⊝ very low1,2,4	-0.01 (-0.16,0.13) ⊕⊕⊕⊝moderate4	GLPG1690		
**-0.17 (-0.32,-0.01)** ⊕⊝⊝⊝very low1,2,4	-0.14 (-0.31,0.03) ⊕⊕⊝⊝low1,4	**-0.13 (-0.25,-0.01)** ⊕⊕⊕⊝moderate**1**	-0.13 (-0.47,0.21) ⊕⊕⊝⊝low1,4	-0.09 (-0.28,0.10) ⊕⊕⊝⊝low1,4	-0.07 (-0.23,0.10) ⊕⊝⊝⊝ very low2,3,4	-0.05 (-0.17,0.08) ⊕⊕⊝⊝low1,4	-0.03 (-0.22,0.15) ⊕⊕⊝⊝low1,4	PRM151	
-0.24 (-0.48,-0.00) ⊕⊕⊝⊝low2,4	-0.21 (-0.46,0.04) ⊕⊕⊕⊝moderate4	-0.20 (-0.42,0.02) ⊕⊕⊕⊕high	-0.20 (-0.59,0.19) ⊕⊕⊕⊝moderate4	-0.16 (-0.42,0.10) ⊕⊕⊕⊝moderate4	-0.14 (-0.39,0.11) ⊕⊝⊝⊝very low1,2,4	-0.12 (-0.34,0.10) ⊕⊕⊕⊝moderate4	-0.11 (-0.36,0.15) ⊕⊕⊕⊝moderate4	-0.07 (-0.32,0.18) ⊕⊕⊝⊝low1,4	Pamrevlumab

Data are MD(95%Cl)

1:Certainty lowered for imprecision

2:Certainty lowered for individual study risk of bias

3:Certainty lowered two levels for imprecision

4:Certainty lowered for indirectness

GRADE Working Group grades of evidence – High quality: Further research is very unlikely to change our confidence in the estimate of effect; Moderate quality: Further research is likely to have an important impact on our confidence in the estimate of effect and may change the estimate; Low quality: Further research is very likely to have an important impact on our confidence in the estimate of effect and is likely to change the estimate; Very low quality: We are very uncertain about the estimate

**Table 8:** SUCRA ranking of FVC (L) absolute change from baseline

**Treatment**	**SUCRA**	**PrBest**	**MeanRank**
Warfarin	85.2	33.8	2.3
Imatinib	73.2	17.8	3.4
Placebo	72.6	2.4	3.5
Macitentan	61.3	32.9	4.5
Bosentan	54.8	8.2	5.1
N-acetylcysteine	47.1	2.5	5.8
Nintedanib	36.2	0	6.7
GLPG1690	34	1.6	6.9
PRM151	22.7	0.1	8
Pamrevlumab	13	0.7	8.8

Higher values of SUCRA indicate higher values of decline in FVC (L) absolute change from baseline

#### FVC (% predicted) absolute change from baseline

**Fig. 8:**
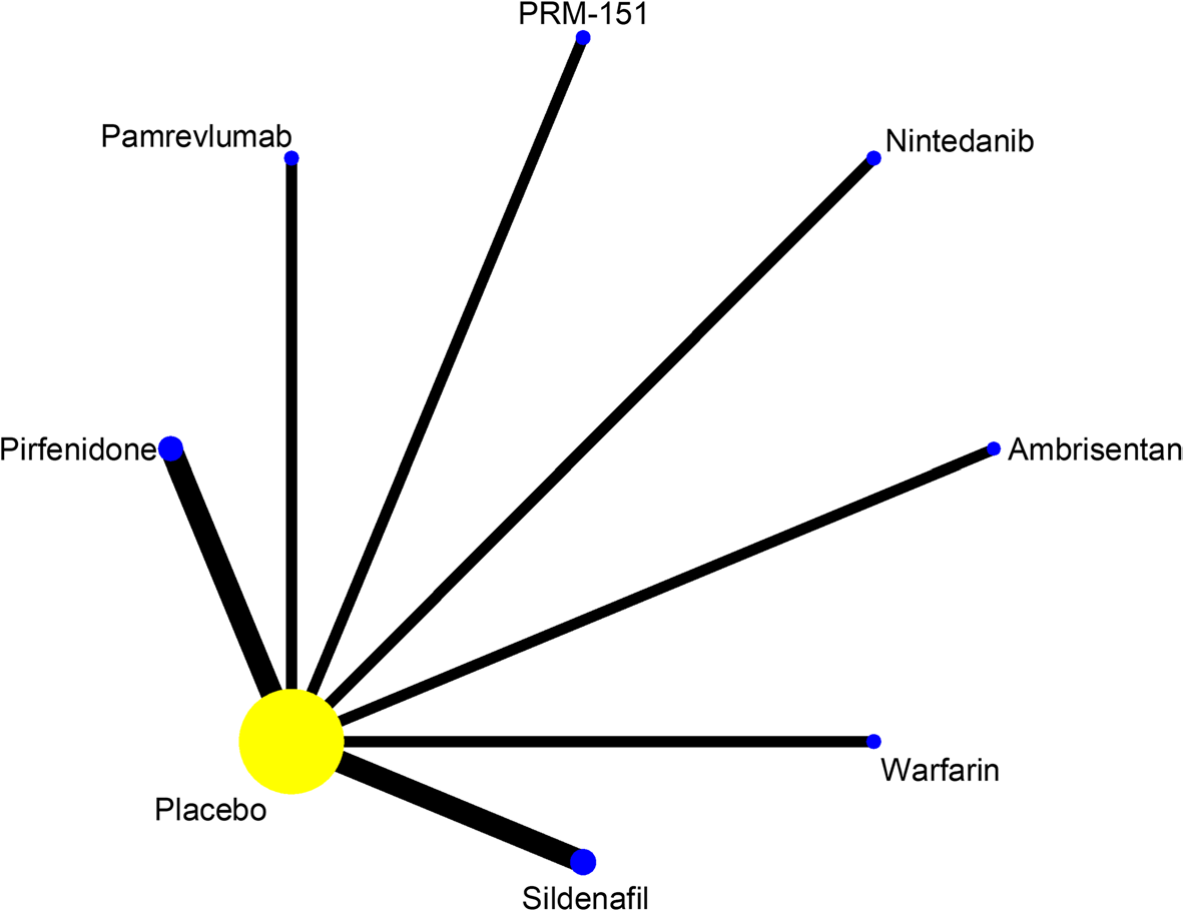
Network evidence map of FVC (% predicted)absolute change from baseline. A total of 9 studies reported the FVC (% predicted) absolute change from baseline in patients with IPF treated with 7 drugs: 1 of Ambrisentan, 1 of Nintedanib, 1 of Pamrevlumab, 2 of Pirfenidone, 1 of PRM-151, 2 of Sildenafil, 1 of Warfarin

Among the 24 included studies, a total of 9 studies reported the FVC (% predicted) absolute change from baseline in patients with IPF treated with 7 drugs, as shown in Fig. 8. The results of the heterogeneity test showed I^2^=42.5% and *P*=0.084, which could be combined for meta-analysis. The consistency model results showed that all study effect sizes (MD) were approximately between -5 and 0, indicating that the consistency of the results was credible (Figure S10 in supplemental content). The results of the NMA (Table 9, Figure S11 in supplemental content) showed no difference in FVC (% predicted)absolute change from baseline improvement with placebo (*P*>0.05) for 7 drugs and so are the comparisons between drugs (*P*>0.05). The SUCRA ranking showed: Ambrisentan (84.9) > Warfarin (79.3) > Placebo(70.3) > Sildenafil(62.5) > Nintedanib(36.3) > Pirfenidone(30.8) > PRM151(19.3) > Pamrevlumab(16.6). Higher values of SUCRA indicate higher values of decline in FVC (% predicted) absolute change from baseline. As shown in Table 10 and Figure S12 in supplemental content.

**Table 9:** Results of network meta-analysis of FVC (% predicted) absolute change from baseline

Ambrisentan							
-1.51 (-8.62,5.60) ⊕⊕⊝⊝low2,4	Warfarin						
-2.40 (-8.73,3.93) ⊕⊕⊕⊝moderate2	-0.89 (-4.14,2.36) ⊕⊕⊕⊝moderate2	Placebo					
-2.79 (-9.59,4.01) ⊕⊕⊝⊝low2,4	-1.28 (-5.37,2.81) ⊕⊕⊝⊝low2,4	-0.39 (-2.88,2.10) ⊕⊕⊕⊕high	Sildenafil				
-4.70 (-11.92,2.52) ⊕⊕⊝⊝low2,4	-3.19 (-7.95,1.57) ⊕⊕⊝⊝low2,4	-2.30 (-5.79,1.19) ⊕⊕⊕⊕high	-1.91 (-6.19,2.37) ⊕⊕⊕⊝moderate4	Nintedanib			
-5.05 (-12.12,2.01) ⊕⊕⊝⊝low2,4	-3.54 (-8.06,0.97) ⊕⊕⊝⊝low2,4	-2.65 (-5.79,0.49) ⊕⊕⊕⊕high	-2.26 (-6.30,1.77) ⊕⊕⊕⊝moderate4	-0.35 (-5.04,4.34) ⊕⊕⊕⊝moderate4	Pirfenidone		
-6.30 (-13.77,1.17) ⊕⊝⊝⊝very low1,2,4	-4.79 (-9.93,0.35) ⊕⊝⊝⊝very low1,2,4	-3.90 (-7.88,0.08) ⊕⊕⊕⊝moderate1	-3.51 (-8.20,1.18) ⊕⊕⊝⊝low1,4	-1.60 (-6.89,3.69) ⊕⊕⊝⊝low1,4	-1.25 (-6.31,3.82) ⊕⊕⊝⊝low1,4	PRM151	
-6.70 (-14.50,1.10) ⊕⊕⊝⊝low2,4	-5.19 (-10.79,0.41) ⊕⊕⊝⊝low2,4	-4.30 (-8.86,0.26) ⊕⊕⊕⊕high	-3.91 (-9.10,1.28) ⊕⊕⊕⊝moderate4	-2.00 (-7.74,3.74) ⊕⊕⊕⊝moderate4	-1.65 (-7.18,3.89) ⊕⊕⊕⊝moderate4	-0.40 (-6.45,5.65) ⊕⊕⊝⊝low1,4	Pamrevlumab

Data are MD(95%Cl)

1:Certainty lowered for imprecision

2:Certainty lowered for individual study risk of bias

3:Certainty lowered two levels for imprecision

4:Certainty lowered for indirectness

GRADE Working Group grades of evidence – High quality: Further research is very unlikely to change our confidence in the estimate of effect; Moderate quality: Further research is likely to have an important impact on our confidence in the estimate of effect and may change the estimate; Low quality: Further research is very likely to have an important impact on our confidence in the estimate of effect and is likely to change the estimate; Very low quality: We are very uncertain about the estimate

**Table 10:** SUCRA ranking of FVC (% predicted) absolute change from baseline

**Treatment**	**SUCRA**	**PrBest**	**MeanRank**
Ambrisentan	84.9	61.5	2.1
Warfarin	79.3	26.8	2.5
Placebo	70.3	2.9	3.1
Sildenafil	62.5	5.9	3.6
Nintedanib	36.3	1.6	5.5
Pirfenidone	30.8	0.5	5.8
PRM151	19.3	0.4	6.7
Pamrevlumab	16.6	0.3	6.8

Higher values of SUCRA indicate higher values of decline in FVC (% predicted) absolute change from baseline

#### The proportion of patients with decline in FVC≥10% predicted

**Fig. 9:**
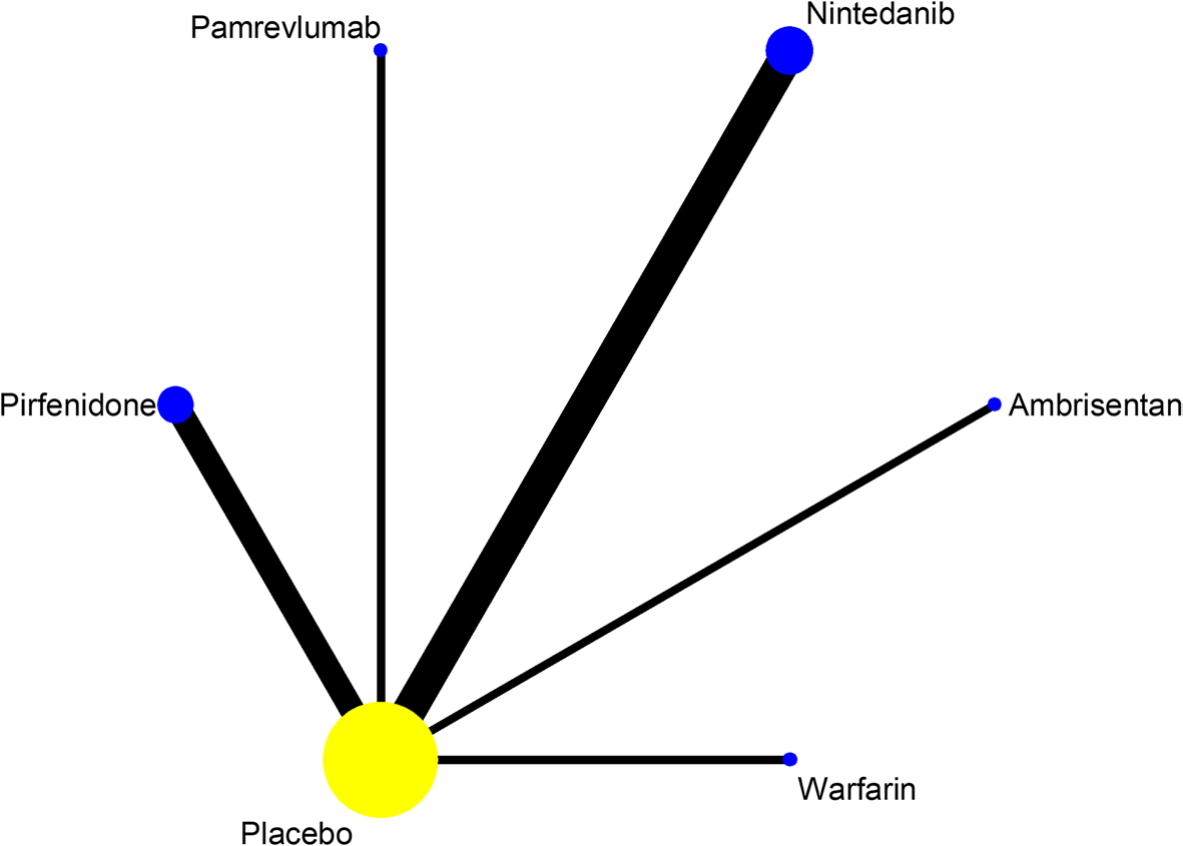
Network evidence map of the proportion of patients with decline in FVC≥10% predicted. A total of 10 studies reported the effect of the proportion of patients with decline in FVC≥ 10% predicted of 5 drugs: 1 of Ambrisentan, 4 of Nintedanib, 1 of Pamrevlumab, 3 of Pirfenidone, 1 of Warfarin

Among the 24 included studies, a total of 10 reported the effect of the proportion of patients with decline in FVC≥ 10% predicted of 5 drugs, as shown in Fig. 9. Statistical analyses were performed by using OR and 95% CI as effect sizes. Although the heterogeneity test results showed that I^2^=62.7%, *P*=0.004, which had some heterogeneity, the consistency model results showed that the log OR of all studies were roughly between 0 and 2, indicating that the consistency of the results was reliable, as shown in Figure S13 in supplemental content. The results of the NMA(Table 11, Figure S14 in supplemental content) showed that Nintedanib (OR=1.81, 95% CI [1.23 to 2.66], high certainty of evidence), Pirfenidone (OR=1.85, 95% CI [1.26 to 2.71], high certainty of evidence), and Pamrevlumab (OR=4.11, 95% CI [ 1.25, 13.58], high certainty of evidence) improved the proportion of patients with the decline in FVC ≥10% predicted better than placebo, and the improvement of the remaining 2 drugs were not different from placebo (*P*> 0.05). the results of comparison between drugs showed: Nintedanib (OR=2.76, 95% CI [1.21 to 6.30], low certainty of evidence), Pirfenidone (OR=2.81, 95%CI [1.23 to 6.42], low certainty of evidence), and Pamrevlumab (OR=6.26, 95% CI [1.54 to 25.40], low certainty of evidence) improved the proportion of patients with decline in FVC ≥10% predicted better than Ambrisentan. The SUCRA ranking showed: Ambrisentan (96) > Placebo(80.4) > Nintedanib(41.8) > Pirfenidone(40.4) > Warfarin(31.3) > Pamrevlumab(10.2). Higher values of SUCRA indicate that the proportion of patients with decline in FVC≥ 10% predicted is higher. As shown in Table 12 and Figure S15 in supplemental content.

**Table 11:** Results of network meta-analysis of the proportion of patients with decline in FVC≥10% predicted

Ambrisentan					
1.52 (0.73,3.16) ⊕⊕⊕⊝moderate2	Placebo				
**2.76 (1.21,6.30)** ⊕⊕⊝⊝low2,4	**1.81 (1.23,2.66)** ⊕⊕⊕⊕high	Nintedanib			
**2.81 (1.23,6.42)** ⊕⊕⊝⊝low2,4	**1.85 (1.26,2.71)** ⊕⊕⊕⊕high	1.02 (0.59,1.76) ⊕⊕⊕⊝moderate4	Pirfenidone		
3.64 (0.81,16.37) ⊕⊕⊝⊝low2,4	2.39 (0.64,8.90) ⊕⊕⊕⊝moderate2	1.32 (0.34,5.19) ⊕⊕⊝⊝low2,4	1.29 (0.33,5.08) ⊕⊕⊝⊝low2,4	Warfarin	
**6.26 (1.54,25.40)** ⊕⊕⊝⊝low2,4	**4.11 (1.25,13.58)** ⊕⊕⊕⊕high	2.27 (0.65,7.96) ⊕⊕⊕⊝moderate4	2.23 (0.64,7.79) ⊕⊕⊕⊝moderate4	1.72 (0.29,10.16) ⊕⊕⊝⊝low2,4	Pamrevlumab

Data are OR(95%Cl)

1:Certainty lowered for imprecision

2:Certainty lowered for individual study risk of bias

3:Certainty lowered two levels for imprecision

4:Certainty lowered for indirectness

GRADE Working Group grades of evidence – High quality: Further research is very unlikely to change our confidence in the estimate of effect; Moderate quality: Further research is likely to have an important impact on our confidence in the estimate of effect and may change the estimate; Low quality: Further research is very likely to have an important impact on our confidence in the estimate of effect and is likely to change the estimate; Very low quality: We are very uncertain about the estimate

**Table 12:** SUCRA ranking of the proportion of patients with decline in FVC≥10% predicted

**Treatment**	**SUCRA**	**PrBest**	**MeanRank**
Ambrisentan	96	84.1	1.2
Placebo	80.4	11.8	2
Nintedanib	41.8	0	3.9
Pirfenidone	40.4	0	4
Warfarin	31.3	3.8	4.4
Pamrevlumab	10.2	0.2	5.5

Higher values of SUCRA indicate that the proportion of patients with decline in FVC ≥ 10% predicted is higher

**Fig. 10:**
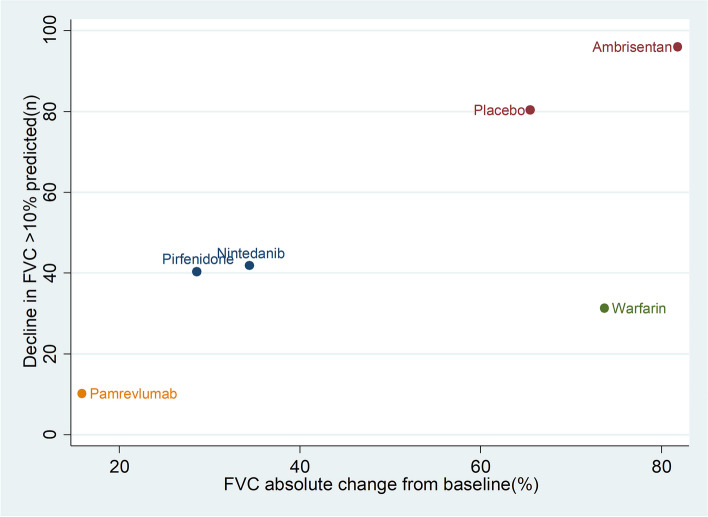
The scatterplot combining the results of FVC (% predicted)absolute change from baseline and the proportion of patients with decline in FVC≥10% predicted(SUCRA values). The horizontal coordinate represents SUCRA values for FVC (% predicted) absolute change from baseline and the vertical coordinate represents SUCRA values for the proportion of patients with decline in FVC ≥10% predicted. If drugs are positioned further to the upper right of the graph, it means that the higher their SUCRA values, the higher their risk; if drugs are positioned further to the lower left of the graph, it means that the lower their SUCRA values are lower, the lower the risk

Combining the results of FVC (% predicted) absolute change from baseline and the proportion of patients with decline in FVC ≥10% predicted, the scatterplot showed (Fig. 10): The SUCRA values of Pamrevlumab, Pirfenidone and Ninedanib were lower than those of placebo, and the SUCRA values of Warfarin and Ambrisentan were higher than those of placebo.

### Publication bias

**Fig. 11:**
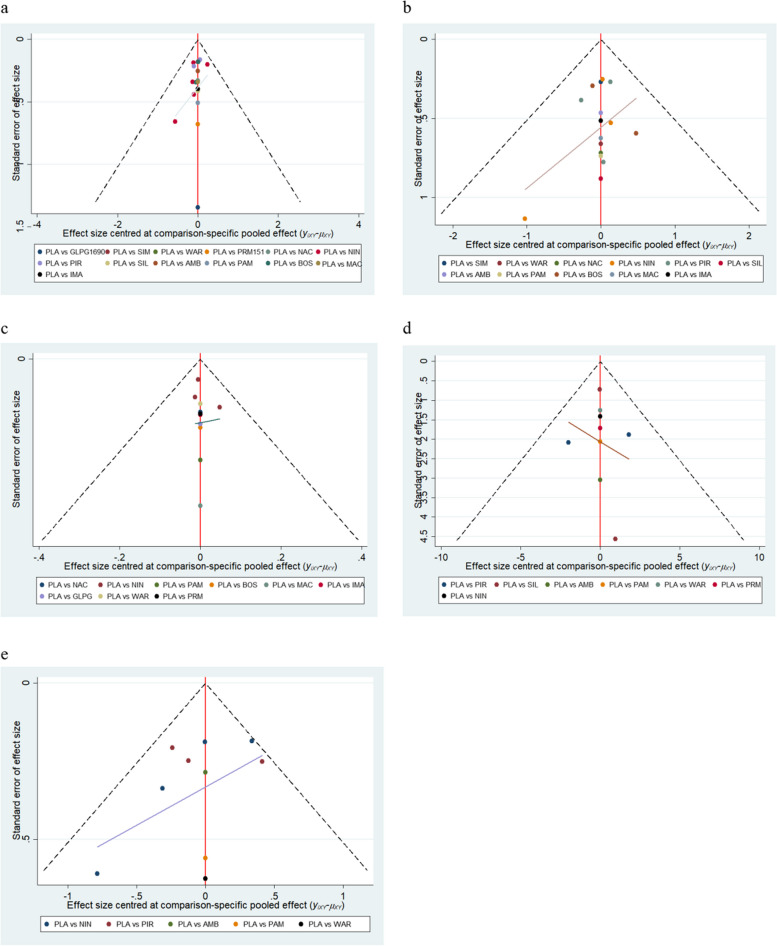
The results of inverted funnel plots. **a** SAEs. **b** All-cause mortality. **c** FVC (L) absolute change from baseline. **d** FVC (% predicted)absolute change from baseline. **e** The proportion of patients with decline in FVC≥10% predicted. (PLA:Placebo; NIN:Nintedanib; PIR:Pirfenidone; SIL:Sildenafil; AMB:Ambrisentan; PAM:Pamrevlumab; BOS:Bosentan; MAC:Macitentan; IMA:Imatinib; GLPG:GLPG1690; SIM:Simtuzumab; WAR:Warfarin;PRM: PRM151;NAC:N-acetylcysteine.)

Figure 11 showed that the inverted funnel plots were symmetrical, suggesting that there’s no publication bias. The statistical results of Begg's test and Egger's test were used to detect bias for all outcomes, and the results showed that P_Begg_>0.05 and P_Egger_> 0.05, indicating that there was no obvious bias in this study. As shown in Figure S16 and Figure S17 in supplemental content.

### Influence analysis

Influence analyses for the 5 outcomes (Table S3-S7 and Figure S18 in supplemental content) showed that none of the included studies had clear sensitivity, indicating that there was no significant difference in the results after excluding any of the studies (except FVC (% predicted) absolute change from baseline). It’s proved that the effect size sensitivity of these outcomes was low, and it had good stability, reliability, and stable and reliable analysis results.

## Discussion

The total of 24 RCTs on the clinical efficacy of 13 drugs for IPF were included in this NMA with the aim of comprehensively assessing their safety and efficacy in the treatment of IPF and differences in this safety and efficacy. Our results found that Nintedanib and Pirfenidone improved lung function (FVC (L) absolute change from baseline or the proportion of patients with the decline in FVC ≥10% predicted) better than placebo, and they improved lung function( FVC (L) absolute change from baseline or the proportion of patients with the decline in FVC ≥10% predicted) better than Warfarin or Ambrisentan. It’s also found that Pirfenidone and Nintedanib had lower all-cause mortality than Warfarin and Ambrisentan. The SUCRA values for the efficacy and safety of Nintedanib and Pirfenidone were also lower than those of placebo and many other drugs. Therefore, Our result confirmed that Nintedanib and Pirfenidone can significantly slow the decline of lung function in IPF patients with better safety profile than placebo and many other drugs.

Cell signaling pathways activated by tyrosine kinases, such as VEGF, FGF, and PDGF, had been shown to be involved in the pathogenesis of IPF [45–47]. Nintedanib (formerly BIBF 1120) is an intracellular inhibitor that targets a variety of tyrosine kinases, including receptors such as VEGF, FGF, and PDGF [48]. Some studies have reported that it can reduce the decline rate of FVC in IPF patients [13, 14]. In a previous phase 2 RCT [TOMORROW], compared with placebo, Nintedanib 150mg bid can better improve the FVC change rate of IPF patients (*P*=0.01), and the incidence of SAEs was lower (27.1% vs.30.6%) [14]. In two repeated RCTs (INPUTLIS-1 and INPUTLIS-2), it also achieved good results, and the incidence of SAEs and all-cause mortality of patients in the Nintedanib group were also lower than those in the placebo group [13]. In addition, similar results have been reported in two recent clinical trials [35, 36]. Pifenidone is an orally bioavailable synthetic molecule. It regulates the activity of TGF-β and TNF-α [49–53], and can inhibit collagen synthesis and fibroblast proliferation [50, 53–56]. In one trial, there was a statistically significant difference in VC decline at 9 months between placebo (-0.13 L) and Pirfenidone (-0.03 L) (*p* = 0.0366). One of the five patients in the placebo group died after an exacerbation episode while there were no deaths in the Pirfenidone group during the 9-month study period, and no SAEs were reported in the Pirfenidone group [15]. One literature later reported on two simultaneous studies (CAPACITY 004 and 006): two studies combined showed the effect of Pirfenidone treatment on predicted percentage FVC at week 72 (*p*=0.005): -8.5% in the Pirfenidone 2403 mg/day group and -11.0% in the placebo group. In terms of security, the rates of SAEs and mortality were lower in the group with Pirfenidone than in the placebo group [37]. In addition, similar results have been reported in two clinical trials [12, 38]. All these findings showed that Nintedanib and Pirfenidone had good effect on slowing the decline of lung function in patients with IPF and had lower incidence of SAEs and all-cause mortality. Therefore, Nintedanib and Pirfenidone can continue to be vigorously promoted in clinical practice.

It’s also found that Pamrevlumab improved the proportion of patients with decline in FVC≥10% predicted better than placebo and Ambrisentan, and it had lower all-cause mortality than Warfarin. In addition, the SUCRA values for the lung function and all-cause mortality of Pamrevlumab were lower than placebo and all other drugs, but the SUCRA value of the incidence of SAEs was higher than placebo. Pamrevlumab (FG-3019) is a fully human recombinant monoclonal antibody against CTGF [57]. In the mice with pulmonary fibrosis, Pamrevlumab improved lung function and lung airway remodeling, and inhibited collagen production [58, 59]. In an open-label study, 89 patients with IPF were given two doses of Pamrevlumab every 3 weeks for 48 weeks, and good results were shown in lung function and quantitative HRCT changes [60]. In another phase 2 RCT, Pamrevlumab can delay the decline rate of the predicted FVC percentage of patients with IPF at 48^th^ week (Pamrevlumab:–2.9% vs. Placebo:–7.2% ). Of the 3 (6%) deaths in the Pamrevlumab group and 6 (11%) deaths in the placebo group, none were considered as treatment-related. However, a higher proportion of treatment-induced urgent SAEs occurred in the Pamrevlumab group than in the placebo group (Pamrevlumab: 24% vs. Placebo: 15%) [18]. Our study confirmed that Pamrevlumab had a very good slowing effect in the declining of lung function and low all-cause mortality in patients with IPF compared to other drugs, and it’s currently in Phase 3 development and may become an important drug for the treatment of IPF in the future. However, it’s also confirmed in our study that the incidence of SAEs is still relatively high, therefore it should be used clinically according to the actual situation of patients.

The results showed no difference in lung function improvement with placebo for Imatinib (FVC (L) absolute change from baseline) and Sildenafil (FVC (% predicted) absolute change from baseline) , and the SUCRA values were also roughly equal to placebo. It’s also found that Imatinib and Sildenafil had lower all-cause mortality than Warfarin, and the SUCRA values for the safety of Imatinib and Sildenafil were also lower than those of placebo and some other drugs. Sildenafil is a phosphodiesterase-5 inhibitor that has been approved by the U.S. Food and Drug Administration for the treatment of idiopathic pulmonary hypertension (IPAH) [61]. It stabilizes cyclic guanosine monophosphate, the second messenger of nitric oxide, which leads pulmonary vasodilation in patients with IPF and thereby improve gas exchange [62]. In addition, sildenafil also reduced the production of superoxide in the mouse model of pulmonary fibrosis [63]. A previous study reported that Sildenafil (20–50 mg orally 3 times daily for 3 months) resulted in an improvement in 6-MWT distances of patients with IPF [64]. However, in one RCT, there was no difference in FVC (% predicted) changes in 89 patients with advanced IPF in the Sildenafil group compared with 91 patients in the placebo group (mean change=0.32, 95% CI [−1.12 to 1.76], *P*=0.66), and there was also no significant difference in SAEs and all-cause mortality [19]. In another RCT, there was no significant difference in FVC (% predicted) in IPF patients (*P*=0.79) between Sildenafil and placebo, and there were few SAEs [20]. Imatinib was approved by the U.S. Food and Drug Administration in 2001 for the treatment of chronic myeloid leukemia and has proven to be very effective. It is a TKI with activity against the PDGFR, DDR, c-kit and c-Abl [65, 66]. Current studies have demonstrated that imatinib inhibits bleomycin-induced IPF [67, 68], and the mechanism of action is closely related to inhibition of lung FMT and inhibition of the ECM produced by PDGF and TGF-β signaling [69]. One phase 2 RCT reported no significant difference between Imatinib and placebo at 96 week’s follow-up in terms of time to disease progression (predicted 10% reduction in FVC percentage from baseline) or time to death, and there was no significant difference in SAEs and mortality [21]. Thus, Sildenafil and Imatinib appear to have lower rates of SAEs and all-cause mortality, but do not have much effect in improving FVC in patients. Our study confirmed that Sildenafil and Imatinib had a better safety profile than other drugs, but their efficacy was less obvious than placebo, so further clinical studies are needed to confirm their effectiveness.

It’s found that PRM151 improved FVC (L) absolute change from baseline in patients with IPF better than placebo and Warfarin. In addition, the SUCRA values for the lung function (FVC (L) absolute change from baseline or FVC (% predicted) absolute change from baseline) and SAEs of PRM151 were lower than placebo and most other drugs. Current clinical study had found very low plasma concentrations of Pentraxin 2 in patients with IPF [70] and had demonstrated in experimental studies that PRM-151 (Recombinant human pentraxin-2) inhibited TGF-β1 and bleomycin-induced pulmonary fibrosis [70–72]. Its mechanism of improving pulmonary fibrosis was closely related to inhibition of TGF-β1 production and inhibition of monocytes differentiation into pro-inflammatory macrophages and prefibrous fibroblasts [73–75]. In a phase 1 RCT of increasing doses of PRM-151, the results showed a trend towards improvement in FVC and 6-MWT in the combined dose group of PRM-151 and no SAEs [43]. In another phase 2 RCT, PRM-151 was also found to improve FVC from baseline to week 28 as a percentage of predicted value (difference, +2.3 [90% CI, 1.1 to 3.5], *P* = 0.001) in patients with IPF, and a lower incidence of SAEs [22]. Therefore, PRM151 appears to improve lung function well in patients with IPF, as well as a low incidence of SAEs, and our findings confirmed these views, and it may be a good targeted new drug for the treatment of IPF. However, due to the lack of data on all-cause mortality, further clinical studies are needed to confirm its safety and efficacy.

The results showed no difference in FVC (L)absolute change from baseline improvement with placebo for GLPG1690, but it had lower SUCRA values than placebo and many other drugs. It’s also found that GLPG1690 had lower incidence of SAEs than Warfarin, and GLPG1690 had lower SUCRA values than placebo and all other drugs. GLPG1690 is a potent and selective autoclassifier protein inhibitor and is well tolerated orally in humans [76, 77]. The results showed that GLPG1690 could improve the Ashcroft fibrosis score of mice with pulmonary fibrosis well and inhibit the profibrotic mediator in IPF fibroblasts [76, 78, 79]. In a phase 2a RCT, patients with IPF received either placebo (*n*=6) or oral GLPG1690 600 mg (*n*=7) once daily for 12 weeks, and the results showed that at week 12, patients in the GLPG1690 group had an average change in FVC of 25 mL versus -70 mL of placebo. SAEs occurred in 2 patients in the placebo group and 1 in the GLPG1690 group, and no patients died [23]. Combined with our findings, these data showed that although GLPG1690 had a low incidence of SAEs and had a certain effect on improving lung function, the patient sample size was too small, and there’s a lack of data on all-cause mortality, therefore further clinical studies are needed to confirm its safety and efficacy.

The results showed no difference in FVC (L)absolute change from baseline improvement with placebo for N-acetylcysteine (NAC), but it had lower SUCRA values than placebo and some other drugs. It’s also found that there’s no difference in the incidence of SAEs and all-cause mortality of N-acetylcysteine treated with IPF compared to placebo. In addition, the SUCRA values for the incidence of SAEs and all-cause mortality of NAC were also higher than those of placebo and many other drugs. Current studies had shown that NAC was able to directly scavenge oxygen radicals [80], and can inhibit TGF-β signaling in IPF [81]. No difference in NAC 600 mg tid versus placebo was found in one RCT in improving FVC and mortality in people with IPF [24]. Another multicentre RCT also found no difference in efficacy or safety between inhaled NAC and placebo [44]. In addition, a RCT(IFIGENIA), compared NAC + therapy including Prednisone and Azathioprine with Prednisone + Azathioprine + Placebo, found that NAC group can better improve VC and DLCO in IPF patients [82]. However, another study (PANTHER-IPF ) reported that the simultaneous use of these three drugs increased mortality and the incidence of SAEs in patients with IPF [83]. Our study also showed that NAC alone wasn’t evident in terms of effectiveness and there may be a number of adverse events, thus NAC alone is not recommended clinically for IPF.

Procoagulases may directly stimulate fibrosis through cell surface receptor-mediated responses [84]. A previous unblinded study reported a 1-year survival benefit with anticoagulation (Heparin + Warfarin) in patients with IPF [85]. However, in a later RCT, warfarin was associated with an increase in all-cause mortality (14 warfarin versus 3 Placebo deaths; *P* = 0.005), and the study had to be terminated early due to excessive mortality [25]. Data from preclinical models suggested that the expression of endothelin receptors in IPF lung tissue increased while antagonizing endothelin receptors may reduce the severity of pulmonary fibrosis [86, 87]. Ambrisentan is a selective ETA receptor antagonist that had been approved for treatment of pulmonary arterial hypertension(PAH) [88]. One RCT reported that Ambrisentan treatment for IPF increased mortality (Ambrisentan: 7.9% vs. Placebo: 3.7%) and an increase in the proportion of patients with IPF with decreased lung function (Ambrisentan: 16.7% vs. Placebo: 11.7%). As a result, the study was terminated early [26]. Our findings also confirmed that the safety and efficacy of Warfarin and Ambrisentan were very poor compared to other drugs and placebo. Combing with previous findings that Warfarin and Ambrisentan should not be used clinically for IPF. In addition, several other drugs (Bosentan, Macitentan and Simtuzumab) are less outstanding than placebo in terms of safety and efficacy, and further clinical studies are needed to confirm their efficacy.

Limitations of Inclusion: The 24 studies we included described the outcomes of the experimental group and the control group in detail, but there are some problems remained:1) All the 24 studies reported on randomization, but 5 studies only mentioned randomization and did not give a clear randomization method; 2 studies did not report allocation concealment and 1 did not use blinding. These factors will influence the overall quality of the study to some extent; 2) The evaluation outcomes of the 24 studies were not exactly the same, and the criteria for evaluating the results were not exactly the same, so the results of this study may have some heterogeneity and sensitivity; 3) There were some small studies in the included studies, and there was some clinical heterogeneity (different doses administered, different administration methods, different courses of treatment, different course of disease, different interventions and different disease severity, etc.), which all affected the reliability of the results; 4) Some studies did not perform statistical analysis of important observation outcomes such as FEV1%, FVC%, TLC%, 6MWD, SGRQ, HRCT, inflammatory factors, pulmonary fibrosis factors, etc., which will affect the overall quality of the literature included in the statistics; 5) All the studies were in English instead of other languages.

## Conclusion

Nintedanib and Pirfenidone can significantly slow the decline of lung function in IPF patients and have a better safety profile, they can continue to be vigorously promoted in clinical practice. Pamlumumab has a good slowing effect on lung function decline and low all-cause mortality in IPF patients and is currently in phase 3 development, it may become an important drug for the treatment of IPF in the future. Sildenafil and Imatinib have a good safety profile, but their effectiveness is not obvious, and further clinical studies are needed to confirm their effectiveness. Both PRM151 and GLPG1690 seem to have the effect of improving lung function of IPF patients, and the incidence of SAEs is low. However, due to the lack of data on all-cause mortality, further clinical studies are needed for comprehensive evaluation. N-acetylcysteine alone is not evident in terms of efficacy, and there may be a number of adverse events, so NAC alone is not recommended clinically for IPF. Warfarin and Ambrisentan have poor safety and efficacy, therefore they are not recommended for clinical use in the treatment of IPF.

In addition to the above drugs, some other drugs such as Nalbuphine [89], pembrolizumab [90], and Treprostinil [91] have shown good efficacy in clinical and animal experimental studies in IPF or interstitial lung disease. In the future, if there are high-quality RCTs of these drugs for IPF, they can be included and evaluated comprehensively. In addition, in future network meta-analysis, various molecular biomarkers in the field of precision medicine can be considered as efficacy evaluation indicators [92] to screen out more effective drugs for the treatment of IPF.

### Supplementary Information

**Additional file 1: Figure S1.** The forest plot of consistency test of SAEs. **Figure S2.** Forest plot of pairwise comparison of the incidence of SAEs.  (PLA: Placebo; NIN:Nintedanib; PIR: Pirfenidone; SIL: Sildenafil; AMB: Ambrisentan; PAM: Pamrevlumab; BOS: Bosentan; MAC: Macitentan; IMA: Imatinib; GLPG:GLPG1690; SIM: Simtuzumab; WAR: Warfarin; PRM: PRM151;NAC:N-acetylcysteine.). **Figure S3.** SUCRA ranking chart of the incidence of SAEs. **Figure S4.** The forest plot of consistency test of all-cause mortality. **Figure S5.** Forest plot of pairwise comparison of all-cause mortality, (PLA: Placebo; NIN: Nintedanib; PIR:Pirfenidone; SIL: Sildenafil; AMB: Ambrisentan; PAM: Pamrevlumab; BOS: Bosentan; MAC:Macitentan; IMA: Imatinib; SIM: Simtuzumab; WAR: Warfarin; NAC:N-acetylcysteine.). **Figure S6.** SUCRA ranking chart of all-cause mortality. **Figure S7.** The forest plot of consistency test of FVC (L) absolute change from baseline. **Figure S8.** Forest plot of pairwise comparison of FVC (L) absolute change from baseline. (PLA: Placebo; NIN: Nintedanib; PAM: Pamrevlumab; BOS: Bosentan; MAC: Macitentan; IMA:Imatinib; GLPG:GLPG1690;WAR:Warfarin; PRM: PRM151;NAC:N-acetylcysteine.). **Figure S9.** SUCRA ranking chart of FVC (L) absolute change from baseline. **Figure S10.** The forest plot of consistency test of FVC (% predicted)absolute change from baseline. **Figure S11.** Forest plot of pairwise comparison of FVC (% predicted) absolute change from baseline. (PLA: Placebo; NIN: Nintedanib; PIR: Pirfenidone; SIL: Sildenafil; AMB: Ambrisentan; PAM: Pamrevlumab; WAR:Warfarin; PRM: PRM151.). **Figure S12.** SUCRA ranking chart of FVC (% predicted) absolute change from baseline. **Figure S13.** The forest plot of consistency test of the proportion of patients with decline in FVC≥10% predicted. **Figure S14.** Forest plot of pairwise comparison of the proportion of patients with decline in FVC≥10% predicted.  (PLA:Placebo; NIN: Nintedanib; PIR: Pirfenidone; AMB: Ambrisentan; PAM: Pamrevlumab; WAR:Warfarin.). **Figure S15.** SUCRA ranking chart of the proportion of patients with decline in FVC≥10% predicted. **Figure S16.** The funnel chart of bias generation detected by Begg rank correlation. **Figure S17.** The funnel chart generated by Egger's test. **Figure S18.** Influence analysis results of the five main outcomes. **Table S1.** The search strategy of PubMed. **Table S2.** Quality evaluation of the 24 included studies. **Table S3.** Influence analysis results data of the incidence of SAEs. **Table S4.** Influence analysis results data of all-cause mortality. **Table S5.** Influence analysis results data of FVC (L) absolute change from baseline. **Table S6.** Influence analysis results data of FVC (% predicted)absolute change from baseline. **Table S7.** Influence analysis results data of the proportion of patients with decline in FVC≥10% predicted.

## Data Availability

Data supporting our findings are contained within the manuscript.

## Abbreviations

IPF: Idiopathic Pulmonary Fibrosis
AE: Acute exacerbation
FVC: Forced vital capacity
HRCT: High-resolution computed tomography
RCT: Randomized controlled trial
SAE: Serious adverse effect
NAC: N-acetylcysteine
GRADE: Grading of Recommendations Assessment, Development and Evaluation
NMA: Network meta analysis
MD: Mean difference
OR: Odds ratio
95% CI: 95% confidence interval
VEGF: Vascular endothelial growth factor
FGF: Fibroblast growth factor
PDGF: Platelet-derived growth factor
TGF-β: Transforming growth factor-β
TNF-α: Tumour necrosis factor-α
VC: Vital capacity
CTGF: Connective tissue growth factor
IPAH: Idiopathic pulmonary hypertension
6-MWT: 6-minute walk test
TKI: Tyrosine kinase inhibitor
PDGFR: Platelet-derived growth factor receptors
DDR: Discoidin domain receptors
FMT: Fibroblast to myofibroblast transformation
ECM: Extracellular matrix
DLCO: Carbon monoxide diffusivity
ETA: Endothelin A
PAH: Pulmonary arterial hypertension
