# Supplementary material for: A comprehensive comparison of the safety and efficacy of drugs in the treatment of idiopathic pulmonary fibrosis: a network meta-analysis based on randomized controlled trials
Source: BMC Pulm Med. 2024 Jan 27;24:58. doi: 10.1186/s12890-024-02861-w (PMC10822186; doi:10.1186/s12890-024-02861-w)
Supplement: Supplementary file 1 — Additional file 1: Figure S1. The forest plot of consistency test of SAEs. Figure S2. Forest plot of pairwise comparison of the incidence of SAEs. (PLA: Placebo; NIN:Nintedanib; PIR: Pirfenidone; SIL: Sildenafil; AMB: Ambrisentan; PAM: Pamrevlumab; BOS: Bosentan; MAC: Macitentan; IMA: Imatinib; GLPG:GLPG1690; SIM: Simtuzumab; WAR: Warfarin; PRM: PRM151;NAC:N-acetylcysteine.). Figure S3. SUCRA ranking chart of the incidence of SAEs. Figure S4. The forest plot of consistency test of all-cause mortality. Figure S5. Forest plot of pairwise comparison of all-cause mortality, (PLA: Placebo; NIN: Nintedanib; PIR:Pirfenidone; SIL: Sildenafil; AMB: Ambrisentan; PAM: Pamrevlumab; BOS: Bosentan; MAC:Macitentan; IMA: Imatinib; SIM: Simtuzumab; WAR: Warfarin; NAC:N-acetylcysteine.). Figure S6. SUCRA ranking chart of all-cause mortality. Figure S7. The forest plot of consistency test of FVC (L) absolute change from baseline. Figure S8. Forest plot of pairwise comparison of FVC (L) absolute change from baseline. (PLA: Placebo; NIN: Nintedanib; PAM: Pamrevlumab; BOS: Bosentan; MAC: Macitentan; IMA:Imatinib; GLPG:GLPG1690;WAR:Warfarin; PRM: PRM151;NAC:N-acetylcysteine.). Figure S9. SUCRA ranking chart of FVC (L) absolute change from baseline. Figure S10. The forest plot of consistency test of FVC (% predicted)absolute change from baseline. Figure S11. Forest plot of pairwise comparison of FVC (% predicted) absolute change from baseline. (PLA: Placebo; NIN: Nintedanib; PIR: Pirfenidone; SIL: Sildenafil; AMB: Ambrisentan; PAM: Pamrevlumab; WAR:Warfarin; PRM: PRM151.). Figure S12. SUCRA ranking chart of FVC (% predicted) absolute change from baseline. Figure S13. The forest plot of consistency test of the proportion of patients with decline in FVC≥10% predicted. Figure S14. Forest plot of pairwise comparison of the proportion of patients with decline in FVC≥10% predicted. (PLA:Placebo; NIN: Nintedanib; PIR: Pirfenidone; AMB: Ambrisentan; PAM: Pamrevlumab; WAR:Warfarin.). Figure S15. SU [file 12890_2024_2861_MOESM1_ESM.zip › Supplementary materials.docx]

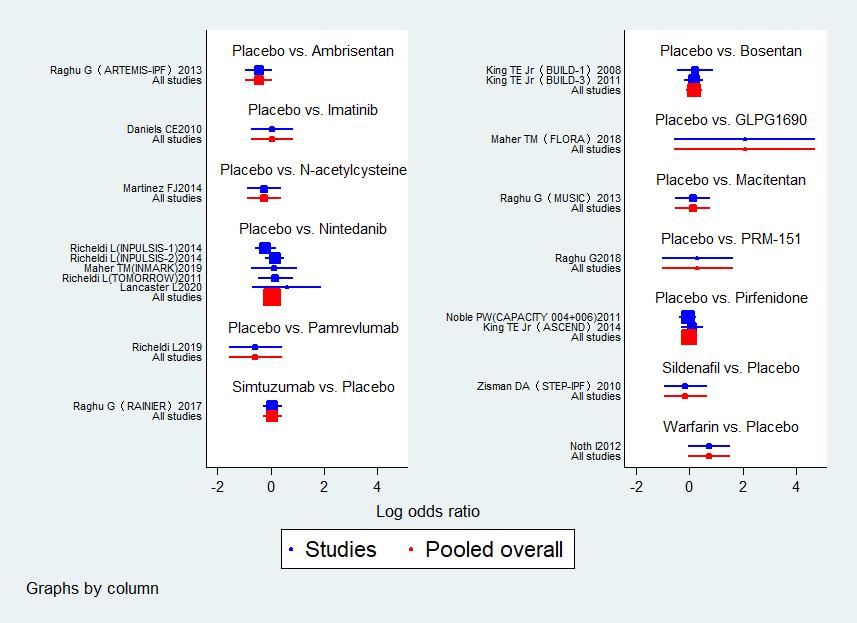


**Figure S1 The forest plot of consistency test of SAEs**


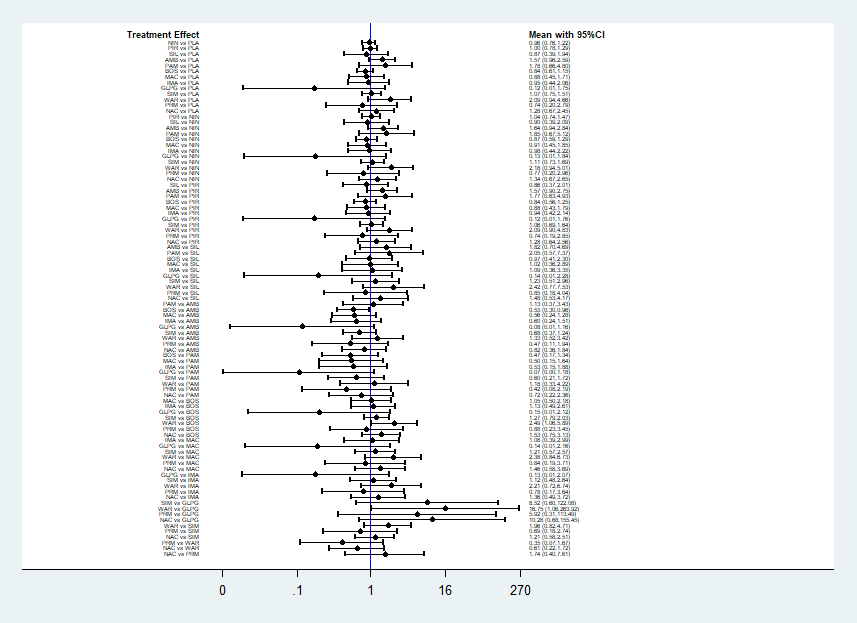


**Figure S2 Forest plot of pairwise comparison of the incidence of SAEs**

(PLA:Placebo; NIN:Nintedanib; PIR:Pirfenidone; SIL:Sildenafil; AMB:Ambrisentan; PAM:Pamrevlumab; BOS:Bosentan; MAC:Macitentan; IMA:Imatinib; GLPG:GLPG1690; SIM:Simtuzumab; WAR:Warfarin;

PRM: PRM151;NAC:N-acetylcysteine.)


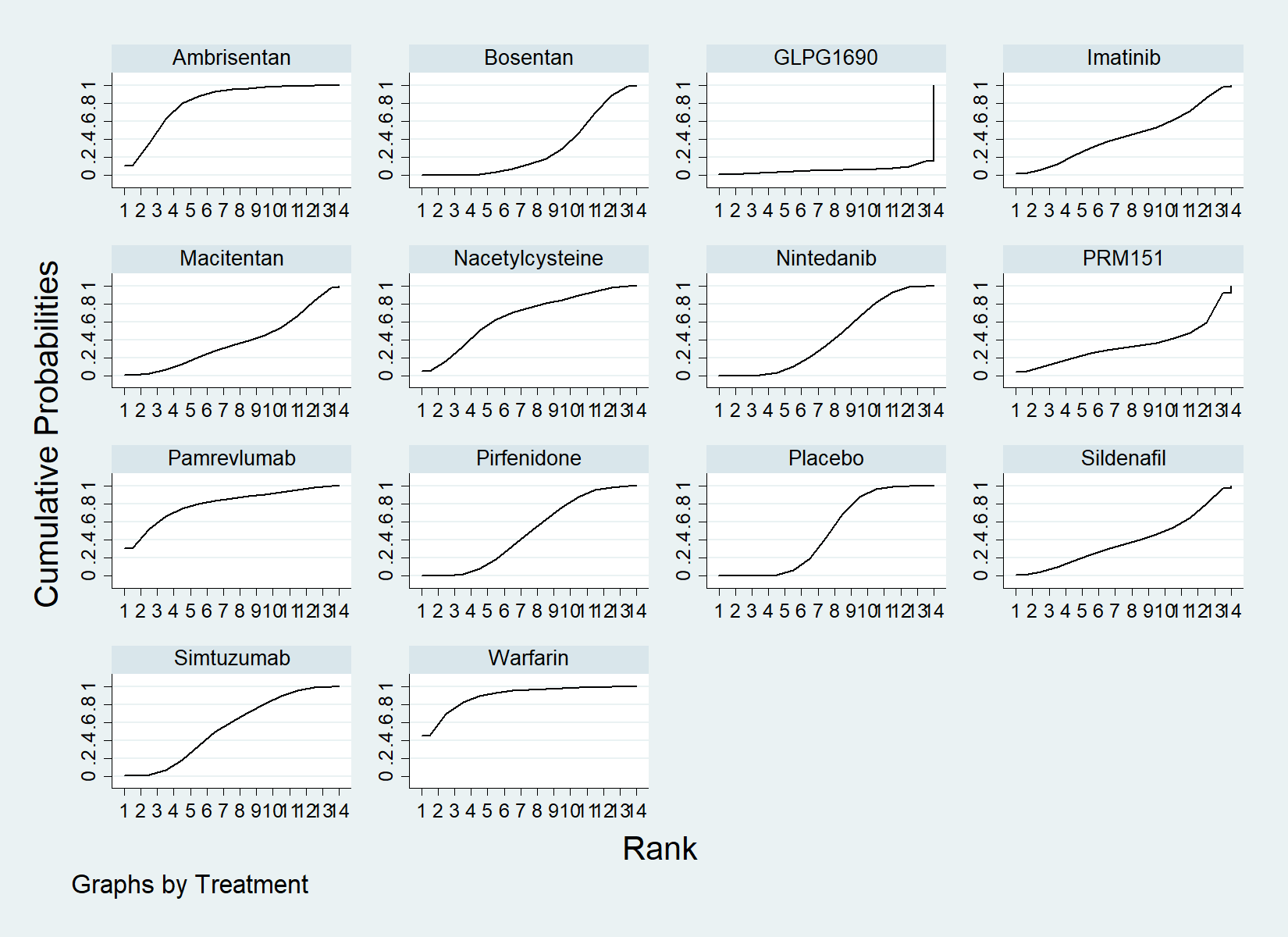


**Figure S3 SUCRA ranking chart of the incidence of SAEs**


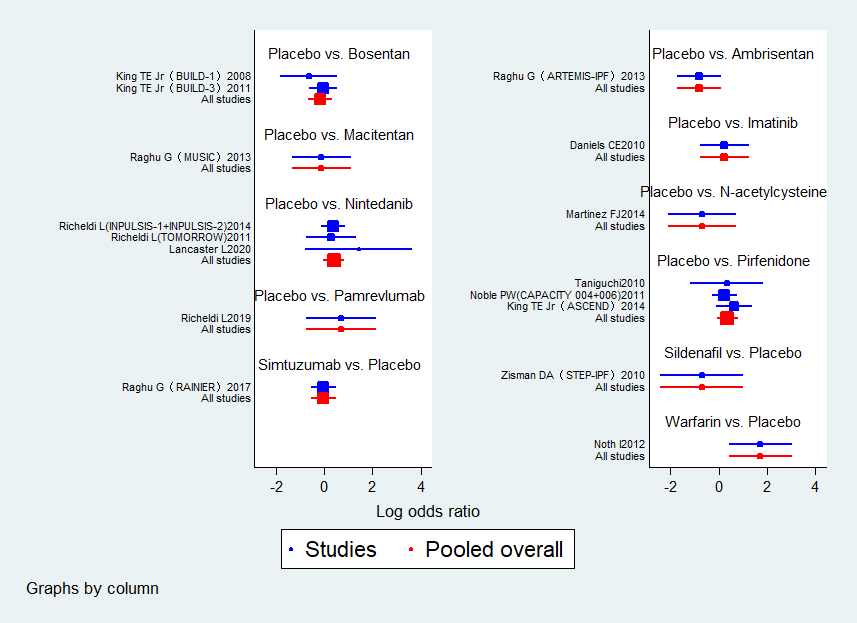


**Figure S4 The forest plot of consistency test of all-cause mortality**

**
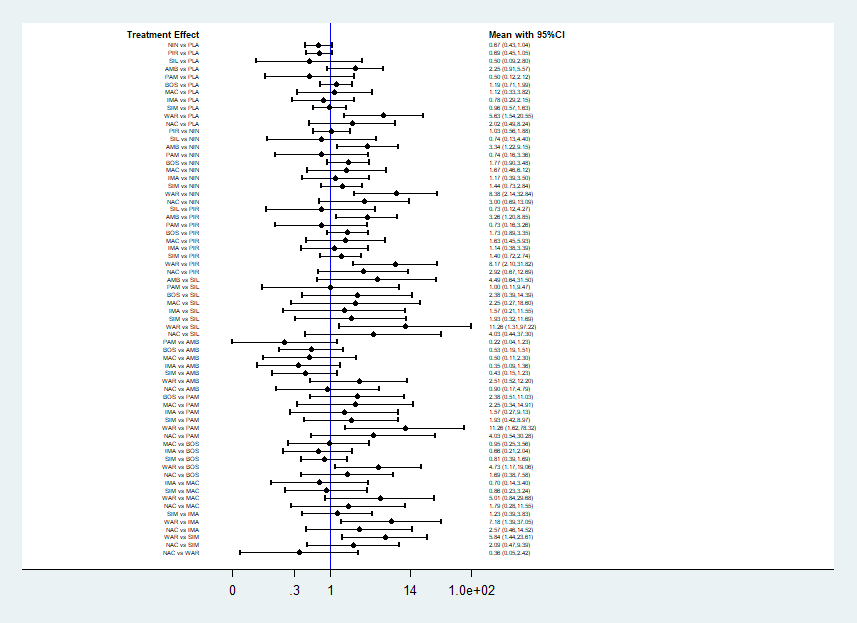
**

**Figure S5 Forest plot of pairwise comparison of all-cause mortality**

(PLA:Placebo; NIN:Nintedanib; PIR:Pirfenidone; SIL:Sildenafil; AMB:Ambrisentan; PAM:Pamrevlumab; BOS:Bosentan; MAC:Macitentan; IMA:Imatinib; SIM:Simtuzumab; WAR:Warfarin; NAC:N-acetylcysteine.)


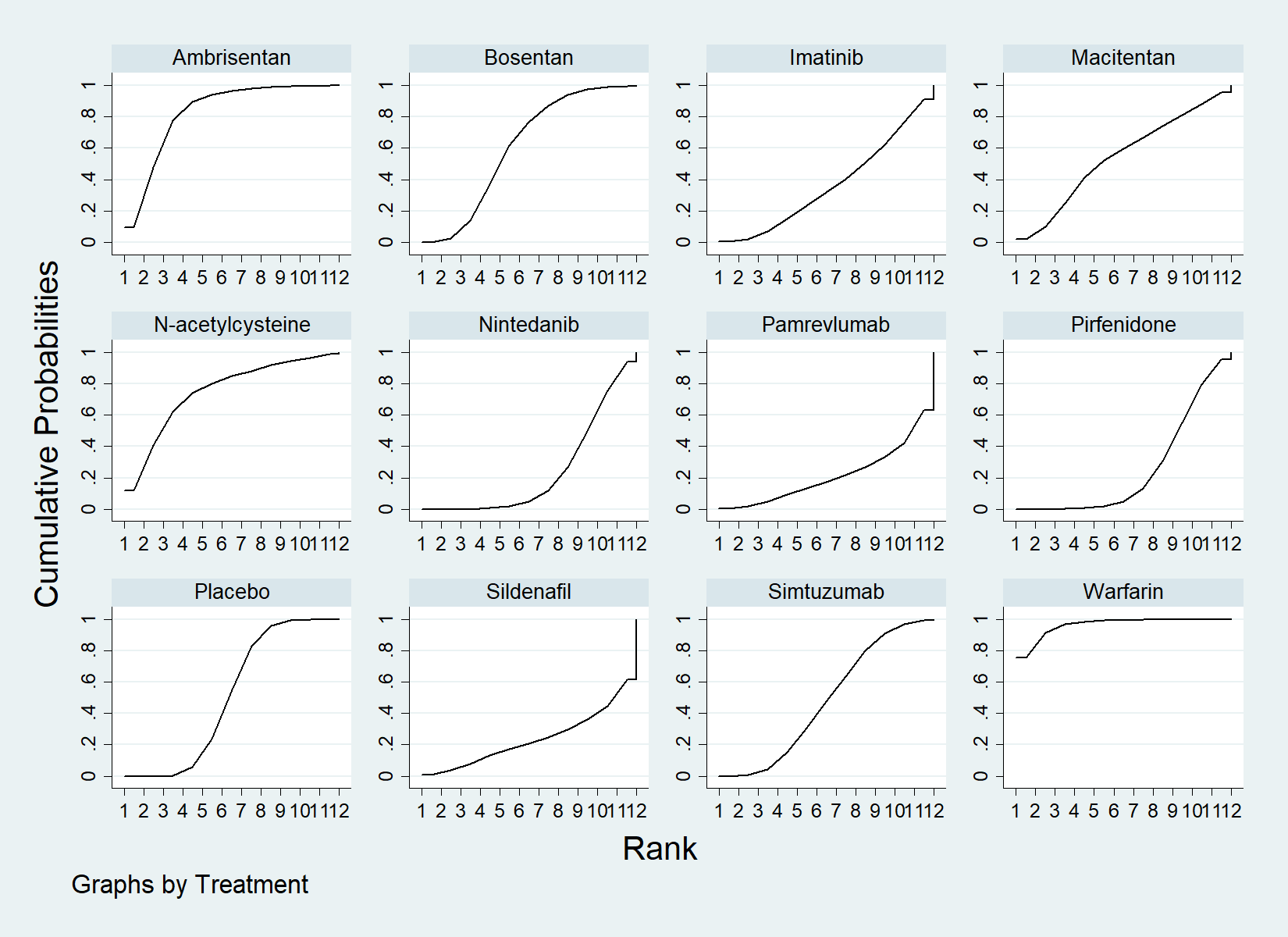


**Figure S6 SUCRA ranking chart of all-cause mortality**


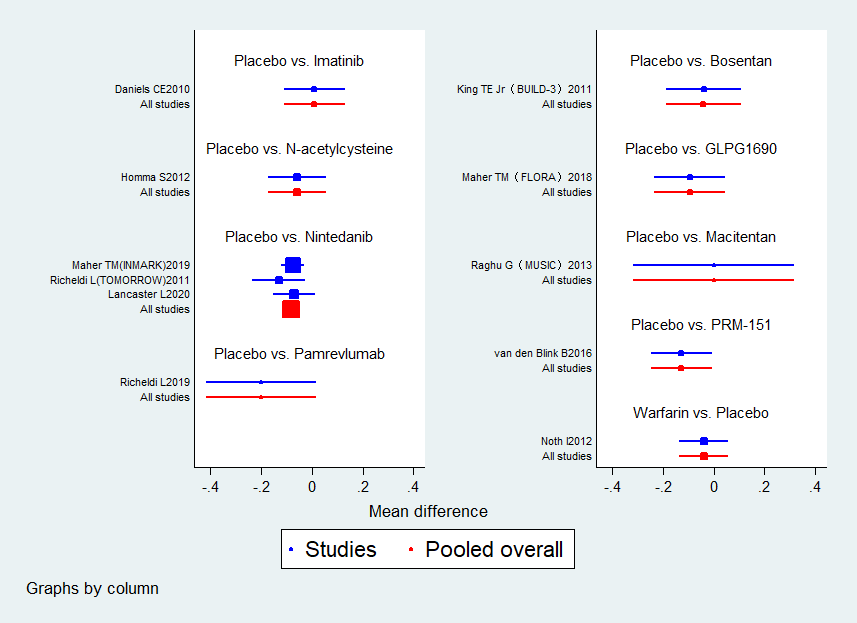


**Figure S7 The forest plot of consistency test of FVC (L) absolute change from baseline**


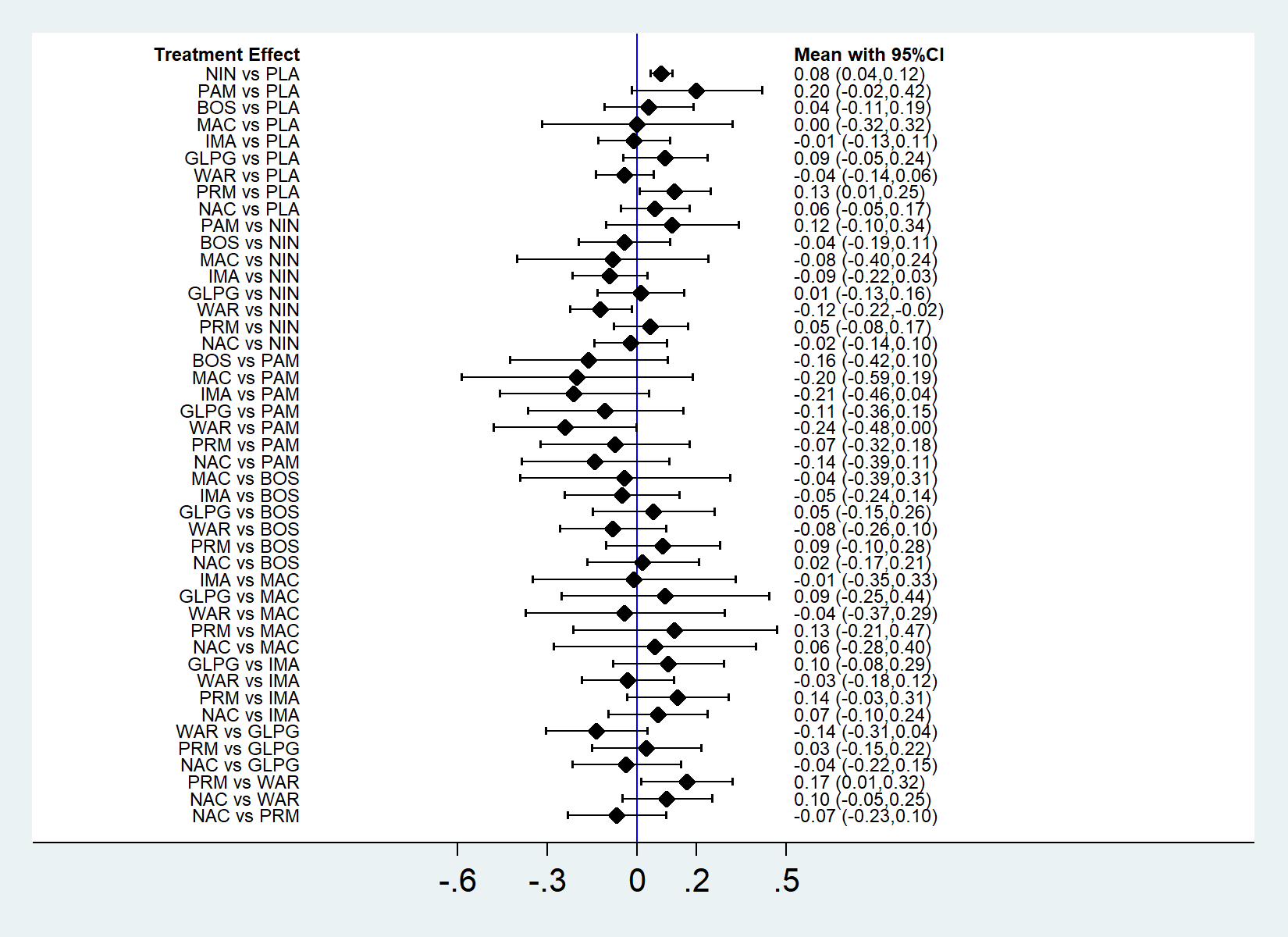


**Figure S8 Forest plot of pairwise comparison of FVC (L) absolute change from baseline**

(PLA:Placebo; NIN:Nintedanib; PAM:Pamrevlumab; BOS:Bosentan; MAC:Macitentan; IMA:Imatinib; GLPG:GLPG1690;WAR:Warfarin;

PRM: PRM151;NAC:N-acetylcysteine.)


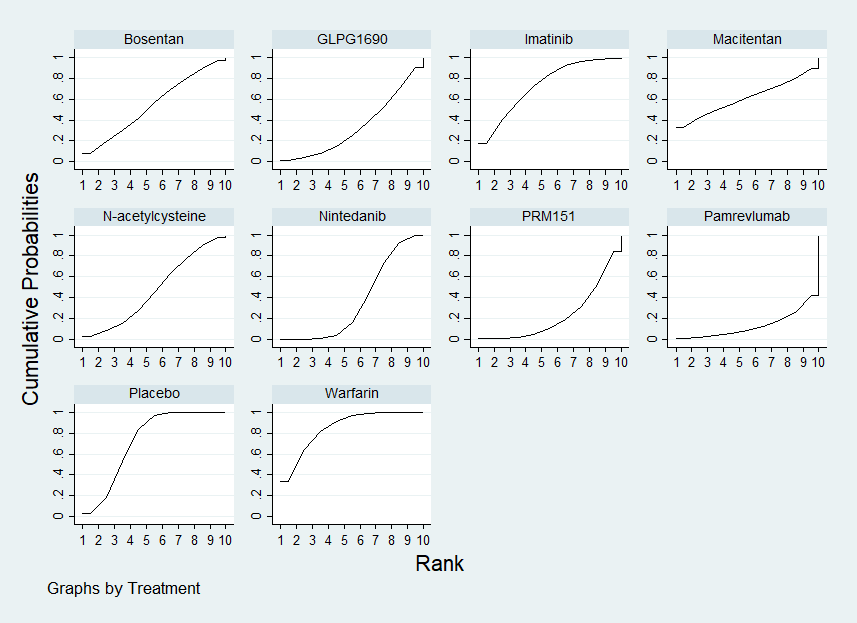


**Figure S9 SUCRA ranking chart of FVC (L) absolute change from baseline**


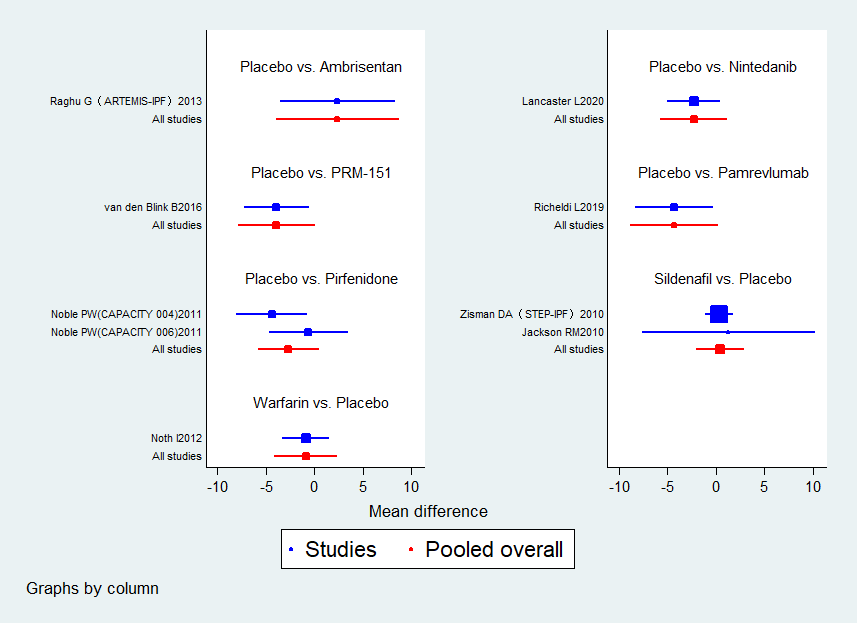


**Figure S10 The forest plot of consistency test of FVC (% predicted)absolute change from baseline**

**
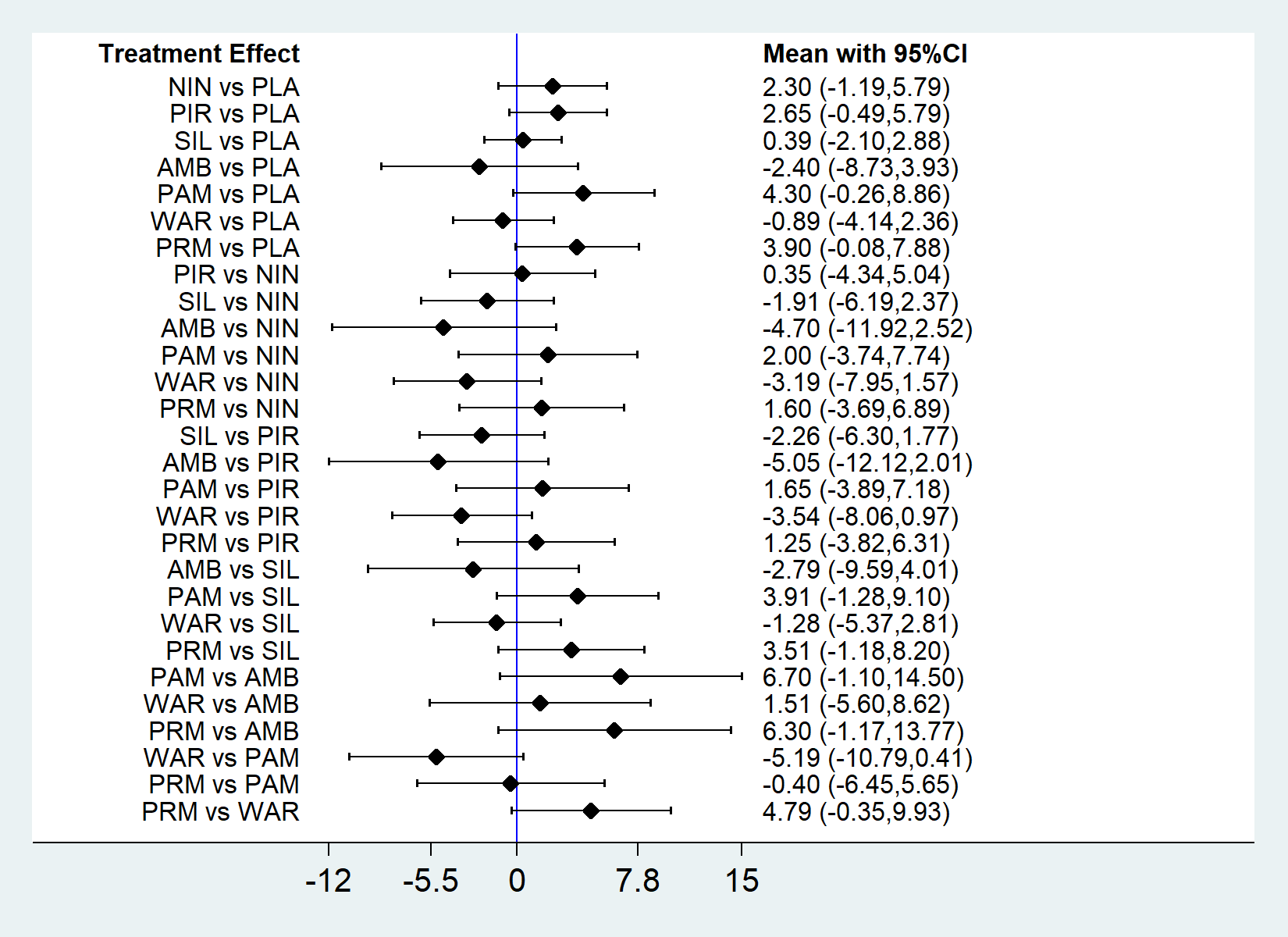
**

**Figure S11 Forest plot of pairwise comparison of FVC (% predicted) absolute change from baseline**

(PLA:Placebo; NIN:Nintedanib; PIR:Pirfenidone; SIL:Sildenafil; AMB:Ambrisentan; PAM:Pamrevlumab; WAR:Warfarin; PRM: PRM151.)


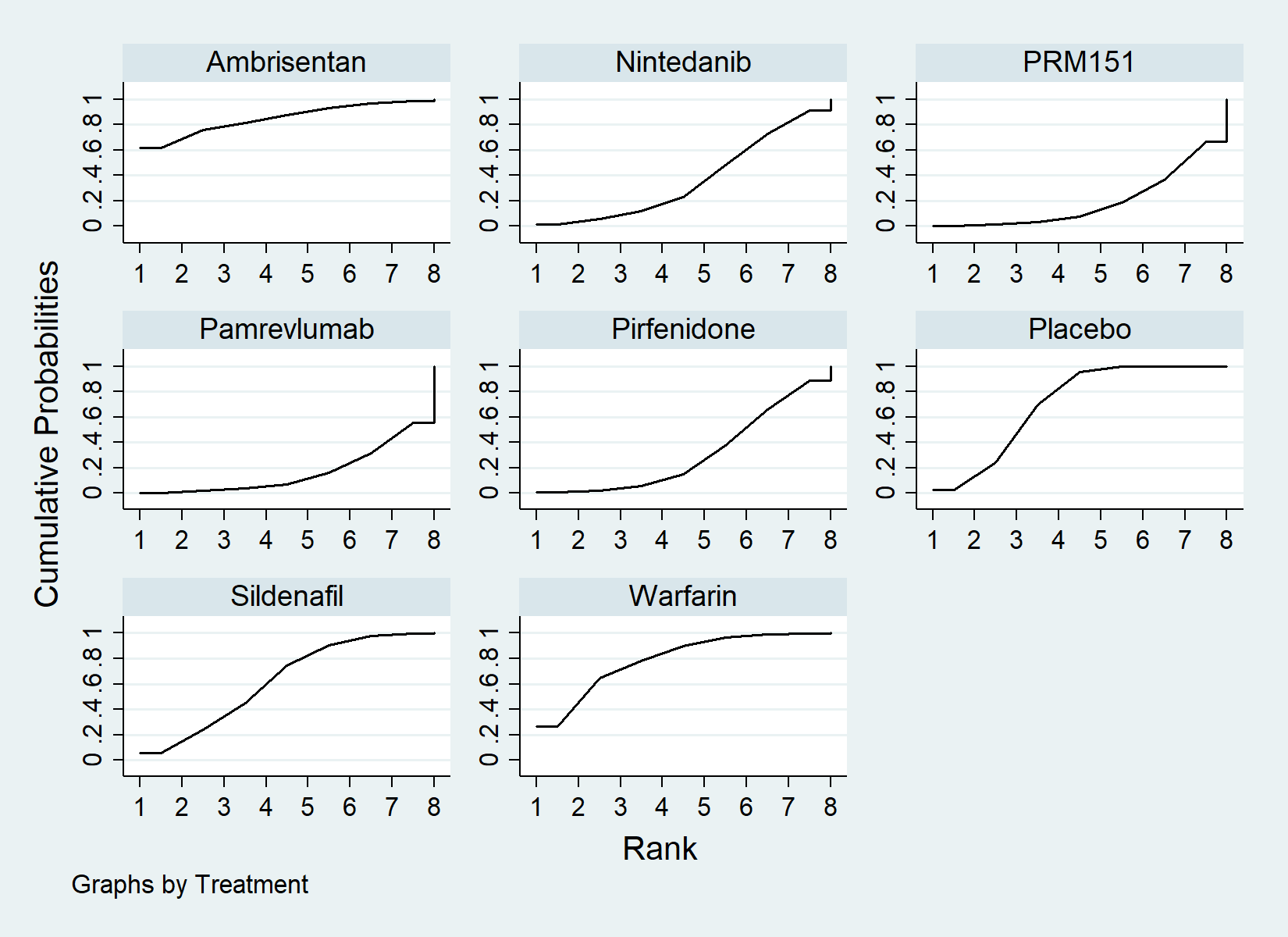


**Figure S12 SUCRA ranking chart of FVC (% predicted) absolute change from baseline**


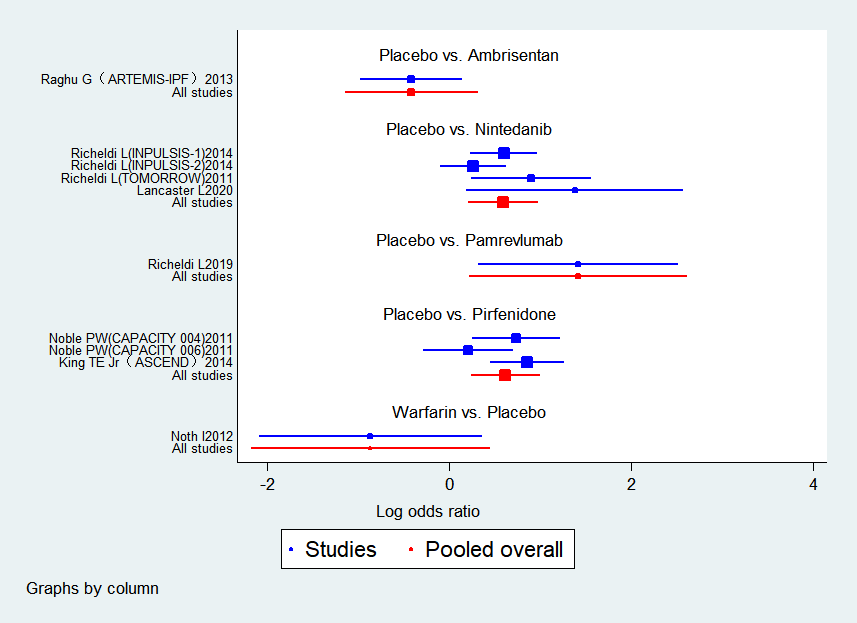


**Figure S13 The forest plot of consistency test of the proportion of patients with decline in FVC≥10% predicted**


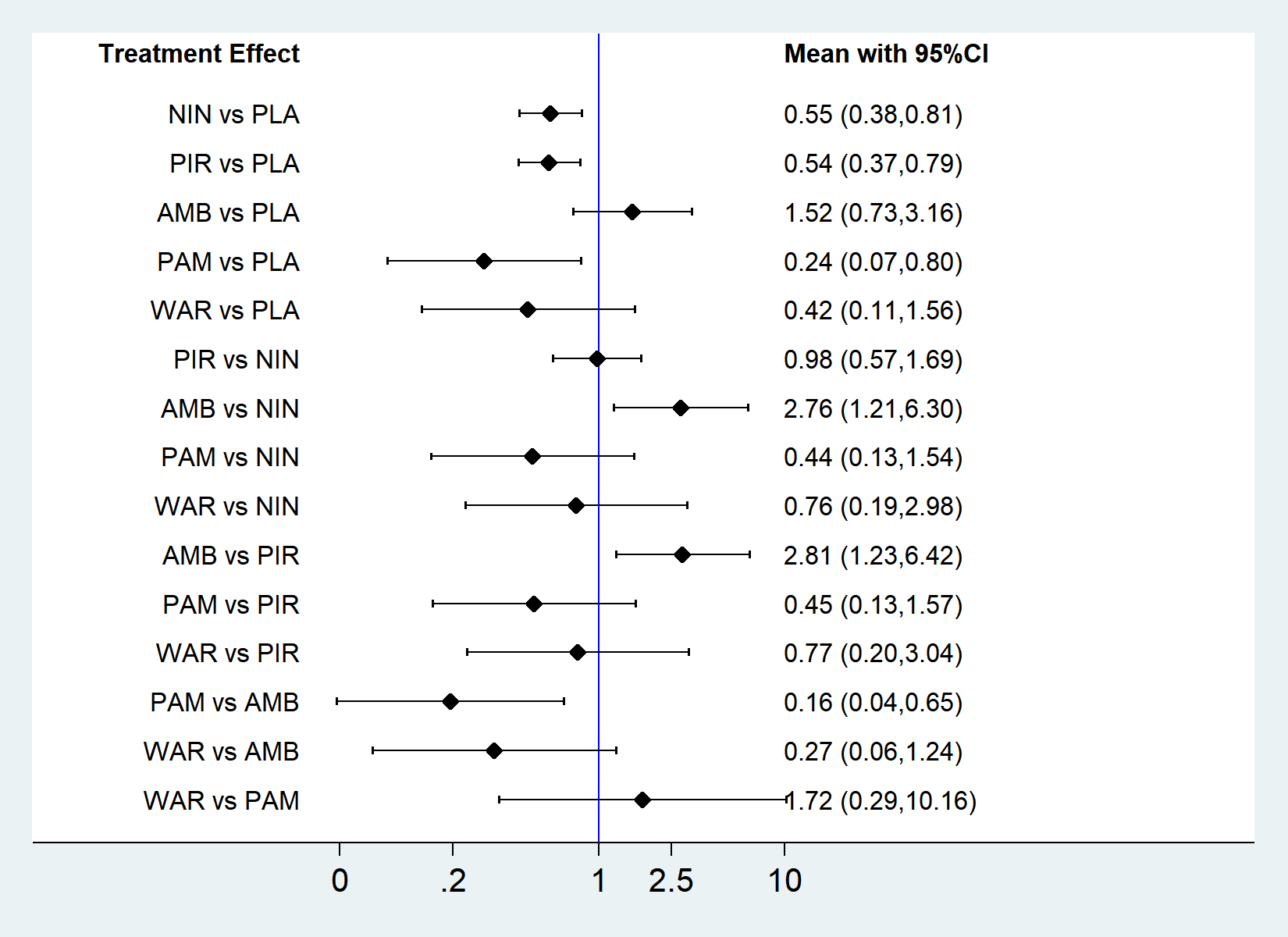


**Figure S14 Forest plot of pairwise comparison of the proportion of patients with decline in FVC≥10% predicted**

(PLA:Placebo; NIN:Nintedanib; PIR:Pirfenidone; AMB:Ambrisentan; PAM:Pamrevlumab; WAR:Warfarin.)


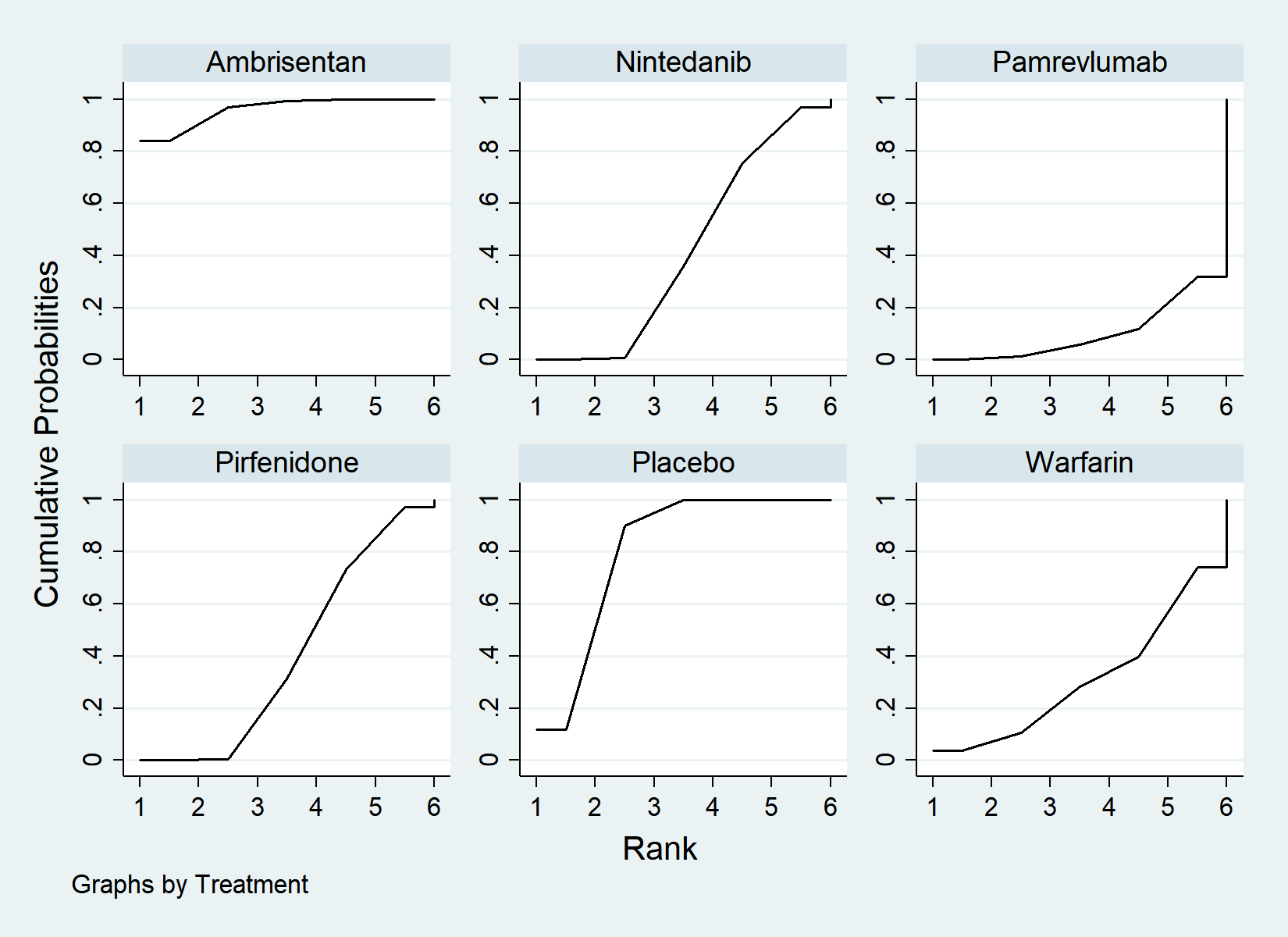


**Figure S15 SUCRA ranking chart of the proportion of patients with decline in FVC≥10% predicted**

| **** | **** |  |
| --- | --- | --- |
| **a.SAEs**  **(Pr > \|z\|=0.529 (continuity corrected))** | **b.All-cause mortality**  **(Pr > \|z\|=0.964 (continuity corrected))** |  |
| **** | **** | **** |
| **c.FVC (L) absolute change from baseline**  **(Pr > \|z\|=0.755 (continuity corrected))** | **d.FVC (% predicted)absolute change from baseline**  **(Pr > \|z\|=0.754 (continuity corrected))** | **e.The proportion of patients with decline in FVC≥10% predicted**  **(Pr > \|z\|=0.474 (continuity corrected))** |

**Figure S16 The funnel chart of bias generation detected by Begg rank correlation**

| **** | **** |  |
| --- | --- | --- |
| **a.SAEs (P>\|t\|=0.488)** | **b.All-cause mortality (P>\|t\|=0.536)** |  |
| **** | **** | **** |
| **c.FVC (L) absolute change from baseline (P>\|t\|=0.955)** | **d.FVC (% predicted)absolute change from baseline (P>\|t\|=0.366)** | **e.The proportion of patients with decline in FVC≥10% predicted (P>\|t\|=0.338)** |

**Figure S17 The funnel chart generated by Egger's test**

|  |  |  |
| --- | --- | --- |
| **a.SAEs** | **b.All-cause mortality** |  |
|  |  |  |
| **c.FVC (L) absolute change from baseline** | **d.FVC (% predicted)absolute change from baseline** | **e.The proportion of patients with decline in FVC≥10% predicted** |

**Figure S18 Influence analysis results of the five main outcomes**

**Table S1 The search strategy of PubMed**

| **Number** | **Search Terms** |
| --- | --- |
| **#1** | Mesh descriptor: (Idiopathic pulmonary fibrosis) explode all trees |
| **#2** | ((((((Pulmonary fibrosisor[Title/Abstract]) OR Pulmonary interstitial fibrosis [Title/Abstract]) OR Interstitial lung disease [Title/Abstract]) OR IPF [Title/Abstract]) |
| **#3** | Or 1-2 |
| **#4** | Mesh descriptor: (medicine) explode all trees |
| **#5** | ((((Drugs[Title/Abstract]) OR treatment[Title/Abstract]) |
| **#6** | Or 4-5 |
| **#7** | Mesh descriptor: (randomized controlled trial) explode all trees |
| **#8** | ((((RCT[Title/Abstract]) OR Clinical trial[Title/Abstract]) |
| **#9** | Or 7-8 |
| **#10** | 6 and 9 |
| **#11** | 3 and 10 |

**Table S2 Quality evaluation of the 24 included studies**

| Studies | Randomization method | Allocation concealment | Blind method | Loss to follow-up | Baseline comparability | Jadad score |
| --- | --- | --- | --- | --- | --- | --- |
| Daniels CE 2010 | Interactive Voice Randomization System | Described | Double-blind | Described | No significant difference | 7 |
| Homma S 2012 | Randomization | No described | No blinding | Described | No significant difference | 3 |
| Jackson RM 2010 | Varying size of the blocks | Described | Double-blind | Described | No significant difference | 7 |
| King TE Jr（ASCEND）2014 | Permuted block design | Described | Double-blind | Described | No significant difference | 7 |
| King TE Jr（BUILD-1）2008 | Randomization | Described | Double-blind | Described | No significant difference | 6 |
| King TE Jr（BUILD-3）2011 | Randomization | Described | Double-blind | Described | No significant difference | 6 |
| Lancaster L 2020 | Randomization | Described | Double-blind | Described | No significant difference | 6 |
| Maher TM（FLORA）2018 | Permuted block design | Described | Double-blind | Described | No significant difference | 7 |
| Maher TM(INMARK)2019 | Pseudo-random number generator in block sizes | Described | Double-blind | Described | No significant difference | 7 |
| Martinez FJ 2014 | Permuted block design | Described | Double-blind | Described | No significant difference | 7 |
| Noble PW(CAPACITY 004)2011 | Permuted block design | Described | Double-blind | Described | No significant difference | 7 |
| Noble PW(CAPACITY 006)2011 | Permuted block design | Described | Double-blind | Described | No significant difference | 7 |
| Noth I 2012 | Permuted block design | Described | Double-blind | Described | No significant difference | 7 |
| Raghu G 2018 | Software-generated randomization list | Described | Double-blind | Described | No significant difference | 7 |
| Raghu G（ARTEMIS-IPF）2013 | Stratified ran domization | Described | Double-blind | Described | No significant difference | 7 |
| Raghu G（MUSIC）2013 | Centralised system | Described | Double-blind | Described | No significant difference | 7 |
| Raghu G（RAINIER）2017 | Interactive Voice Randomization System | Described | Double-blind | Described | No significant difference | 7 |
| Richeldi L 2019 | Interactive Voice Randomization System | Described | Double-blind | Described | No significant difference | 7 |
| Richeldi L(INPULSIS-1)2014 | Interactive Voice Randomization System | Described | Double-blind | Described | No significant difference | 7 |
| Richeldi L(INPULSIS-2)2014 | Interactive Voice Randomization System | Described | Double-blind | Described | No significant difference | 7 |
| Richeldi L(TOMORROW)2011 | Interactive Voice Randomization System | Described | Double-blind | Described | No significant difference | 7 |
| Taniguchi 2010 | Modified minimisation method | Described | Double-blind | Described | No significant difference | 7 |
| van den Blink B 2016 | Randomization | No described | Double-blind | Described | No significant difference | 5 |
| Zisman DA（STEP-IPF）2010 | Permuted block design | Described | Double-blind | Described | No significant difference | 7 |

**Table S3 Influence analysis results data of the incidence of SAEs**

| Study omitted | Estimate | [95% Conf. Interval] | |
| --- | --- | --- | --- |
| Daniels CE-2010 | 1.0219735 | 0.90384316 | 1.1555432 |
| King TE Jr（ASCEND）-2014 | 1.030925 | 0.90820664 | 1.1702254 |
| King TE Jr（BUILD-1）-2008 | 1.0284297 | 0.90905029 | 1.1634865 |
| King TE Jr（BUILD-3）-2011 | 1.0453187 | 0.91860205 | 1.1895154 |
| Lancaster L-2020 | 1.0260227 | 0.90825719 | 1.1590577 |
| Maher TM (FLORA) -2018 | 1.0249276 | 0.90765536 | 1.1573519 |
| Maher TM (INMARK) -2019 | 1.0233648 | 0.90530992 | 1.1568142 |
| Martinez FJ -2014 | 1.011435 | 0.89389193 | 1.1444345 |
| Noble PW (CAPACITY004+006) -2011 | 1.0129354 | 0.8884297 | 1.1548895 |
| Noth I -2012 | 1.0022742 | 0.88641554 | 1.1332761 |
| Raghu G -2018 | 1.0227677 | 0.90546066 | 1.1552722 |
| Raghu G (ARTEMIS-IPF) -2013 | 0.99073195 | 0.87399375 | 1.1230627 |
| Raghu G (MUSIC) -2013 | 1.0252659 | 0.90624934 | 1.1599128 |
| Raghu G (RAINIER) -2017 | 1.013999 | 0.89095157 | 1.1540401 |
| Richeldi L-2019 | 1.0113508 | 0.89495194 | 1.1428887 |
| Richeldi L (INPULSIS-1) -2014 | 1.0006167 | 0.88070357 | 1.1368566 |
| Richeldi L (INPULSIS-2) -2014 | 1.0405591 | 0.91505945 | 1.1832707 |
| Richeldi L (TOMORROW) -2011 | 1.0268724 | 0.90764773 | 1.1617578 |
| Zisman DA (STEP-IPF) -2010 | 1.0239372 | 0.90567756 | 1.1576389 |
| Combined | 1.0200843 | 0.90353783 | 1.1516641 |

**Table S4 Influence analysis results data of all-cause mortality**

| Study omitted | Estimate | [95% Conf. Interval] | |
| --- | --- | --- | --- |
| Daniels CE -2010 | 0.94483685 | 0.76911777 | 1.1607022 |
| King TE Jr (ASCEND) -2014 | 0.98252726 | 0.79641092 | 1.2121378 |
| King TE Jr (BUILD-1) -2008 | 0.91636229 | 0.74662977 | 1.1246804 |
| King TE Jr (BUILD-3) -2011 | 0.9216854 | 0.7430653 | 1.1432427 |
| Lancaster L -2020 | 0.9519856 | 0.77717882 | 1.1661108 |
| Martinez FJ -2014 | 0.92156625 | 0.75158137 | 1.1299965 |
| Noble PW (CAPACITY 004+006) -2011 | 0.96760058 | 0.77781099 | 1.2036997 |
| Noth I -2012 | 0.8792302 | 0.71562213 | 1.080243 |
| Raghu G (ARTEMIS-IPF) -2013 | 0.8862257 | 0.71953583 | 1.0915314 |
| Raghu G (MUSIC) -2013 | 0.93290102 | 0.76041657 | 1.1445099 |
| Raghu G (RAINIER) -2017 | 0.9331848 | 0.7502175 | 1.1607753 |
| Richeldi L -2019 | 0.95043391 | 0.77519733 | 1.1652833 |
| Richeldi L (INPULSIS-1+INPULSIS-2) -2014 | 0.99780965 | 0.79988611 | 1.2447073 |
| Richeldi L (TOMORROW) -2011 | 0.94525576 | 0.76962489 | 1.1609662 |
| Taniguchi -2010 | 0.94241434 | 0.76896793 | 1.1549829 |
| Zisman DA (STEP-IPF) -2010 | 0.94663876 | 0.77261925 | 1.1598532 |
| Combined | 0.93777522 | 0.76659269 | 1.1471833 |

**Table S5 Influence analysis results data of FVC (L) absolute change from baseline**

| Study omitted | Estimate | [95% Conf. Interval] | |
| --- | --- | --- | --- |
| Daniels CE -2010 | 0.07166802 | 0.04187575 | 0.10146029 |
| Homma S -2012 | 0.0674274 | 0.03754098 | 0.09731383 |
| King TE Jr (BUILD-3) -2011 | 0.0680211 | 0.03853479 | 0.09750741 |
| Lancaster L -2020 | 0.06666535 | 0.03576741 | 0.0975633 |
| Maher TM (FLORA) -2018 | 0.06570596 | 0.03614933 | 0.09526259 |
| Maher TM (INMARK) -2019 | 0.06023622 | 0.02214718 | 0.09832526 |
| Noth I -2012 | 0.07744343 | 0.04713875 | 0.1077481 |
| Raghu G (MUSIC) -2013 | 0.06751336 | 0.03847317 | 0.09655356 |
| Richeldi L -2019 | 0.06457534 | 0.03539809 | 0.09375259 |
| Richeldi L (TOMORROW) -2011 | 0.0617517 | 0.03166221 | 0.09184119 |
| vanden Blink B -2016 | 0.06318212 | 0.03336766 | 0.09299658 |
| Combined | 0.06695472 | 0.03803493 | 0.09587451 |

**Table S6 Influence analysis results data of FVC (% predicted)absolute change from baseline**

| Study omitted | Estimate | [95% Conf. Interval] | |
| --- | --- | --- | --- |
| Jackson RM -2010 | 1.1008052 | 0.14710177 | 2.0545087 |
| Lancaster L -2020 | 0.9443655 | -0.06486125 | 1.9535923 |
| Noble PW (CAPACITY 004) -2011 | 0.86897767 | -0.1124311 | 1.8503864 |
| Noble PW (CAPACITY 006) -2011 | 1.1316227 | 0.15673684 | 2.1065085 |
| Noth I -2012 | 1.4491988 | 0.42181921 | 2.4765785 |
| Raghu G (ARTEMIS-IPF) -2013 | 1.1939334 | 0.23338741 | 2.1544793 |
| Richeldi L -2019 | 0.91667914 | -0.05889329 | 1.8922516 |
| vanden Blink B -2016 | 0.86264676 | -0.12559029 | 1.8508838 |
| Zisman DA (STEP-IPF) -2010 | 1.7287447 | 0.45677015 | 3.0007195 |
| Combined | 1.1030466 | 0.15472397 | 2.0513691 |

**Table S7 Influence analysis results data of the proportion of patients with decline in FVC≥10% predicted**

| Study omitted | Estimate | [95% Conf. Interval] | |
| --- | --- | --- | --- |
| King TE Jr (ASCEND) -2014 | 0.64150518 | 0.53809619 | 0.76478684 |
| Lancaster L -2020 | 0.61124384 | 0.5195232 | 0.71915752 |
| Noble PW (CAPACITY 004) -2011 | 0.61737663 | 0.52053553 | 0.73223418 |
| Noble PW (CAPACITY 006) -2011 | 0.57791817 | 0.48744327 | 0.68518627 |
| Noth I -2012 | 0.60402912 | 0.51354444 | 0.71045685 |
| Raghu G (ARTEMIS-IPF) -2013 | 0.54581267 | 0.46044618 | 0.64700609 |
| Richeldi L -2019 | 0.61377287 | 0.52150053 | 0.72237158 |
| Richeldi L (INPULSIS-1) -2014 | 0.61203039 | 0.5119248 | 0.73171133 |
| Richeldi L (INPULSIS-2) -2014 | 0.56352007 | 0.47077182 | 0.67454088 |
| Richeldi L (TOMORROW) -2011 | 0.61533928 | 0.52123576 | 0.72643214 |
| Combined | 0.59992001 | 0.51080051 | 0.70458822 |
